# Decoding the inflammatory signature of the major depressive episode: insights from peripheral immunophenotyping in active and remitted condition, a case–control study

**DOI:** 10.1038/s41398-024-02902-2

**Published:** 2024-06-12

**Authors:** Federico Manuel Daray, Leandro Nicolás Grendas, Ángeles Romina Arena, Vera Tifner, Romina Isabel Álvarez Casiani, Alejandro Olaviaga, Luciana Carla Chiapella, Gustavo Vázquez, Melina Bianca Penna, Fernando Hunter, Cintia Romina Prokopez, Eugenio Antonio Carrera Silva, Andrea Emilse Errasti

**Affiliations:** 1 Instituto de Farmacología, Facultad de Medicina, Universidad de Buenos Aires, Ciudad de Buenos Aires, Argentina; 2 Consejo Nacional de Investigaciones Científicas y Técnicas (CONICET), Ciudad de Buenos Aires, Argentina; 3 Hospital General de Agudos “Dr. Teodoro Álvarez”, Ciudad de Buenos Aires, Argentina; 4 Hospital General de Agudos “Dr. Cosme Argerich”, Ciudad de Buenos Aires, Argentina; 5 Queen’s University Medical School Kingston, Kingston, ON Canada; 6 Hospital General de Agudos “José María Ramos Mejía”, Ciudad de Buenos Aires, Argentina; 7 Hospital Neuropsiquiátrico “Dr. Braulio A. Moyano”, Ciudad de Buenos Aires, Argentina; 8 Instituto de Medicina Experimental (IMEX), Consejo Nacional de Investigaciones Científicas y Técnicas (CONICET), Academia Nacional de Medicina, Ciudad de Buenos Aires, Argentina

**Keywords:** Depression, Diagnostic markers

## Abstract

Depression is a prevalent and incapacitating condition with a significant impact on global morbidity and mortality. Although the immune system’s role in its pathogenesis is increasingly recognized, there is a lack of comprehensive understanding regarding the involvement of innate and adaptive immune cells. To address this gap, we conducted a multicenter case–control study involving 121 participants matched for sex and age. These participants had either an active (or current) major depressive episode (MDE) (39 cases) or a remitted MDE (40 cases), including individuals with major depressive disorder or bipolar disorder. We compared these 79 patients to 42 healthy controls (HC), analyzing their immunological profiles. In blood samples, we determined the complete cell count and the monocyte subtypes and lymphocyte T-cell populations using flow cytometry. Additionally, we measured a panel of cytokines, chemokines, and neurotrophic factors in the plasma. Compared with HC, people endorsing a current MDE showed monocytosis (*p* = 0.001), increased high-sensitivity C-reactive protein (*p* = 0.002), and erythrocyte sedimentation rate (*p* = 0.003), and an altered proportion of specific monocyte subsets. CD4 lymphocytes presented increased median percentages of activation markers CD69^+^ (*p* = 0.007) and exhaustion markers PD1^+^ (*p* = 0.013) and LAG3^+^ (*p* = 0.014), as well as a higher frequency of CD4^+^CD25^+^FOXP3^+^ regulatory T cells (*p* = 0.003). Additionally, patients showed increased plasma levels of sTREM2 (*p* = 0.0089). These changes are more likely state markers, indicating the presence of an ongoing inflammatory response during an active MDE. The Random Forest model achieved remarkable classification accuracies of 83.8% for MDE vs. HC and 70% for differentiating active and remitted MDE. Interestingly, the cluster analysis identified three distinct immunological profiles among MDE patients. Cluster 1 has the highest number of leukocytes, mainly given by the increment in lymphocyte count and the lowest proinflammatory cytokine levels. Cluster 3 displayed the most robust inflammatory pattern, with high levels of TNFα, CX3CL1, IL-12p70, IL-17A, IL-23, and IL-33, associated with the highest level of IL-10, as well as β-NGF and the lowest level for BDNF. This profile is also associated with the highest absolute number and percentage of circulating monocytes and the lowest absolute number and percentage of circulating lymphocytes, denoting an active inflammatory process. Cluster 2 has some cardinal signs of more acute inflammation, such as elevated levels of CCL2 and increased levels of proinflammatory cytokines such as IL-1β, IFNγ, and CXCL8. Similarly, the absolute number of monocytes is closer to a HC value, as well as the percentage of lymphocytes, suggesting a possible initiation of the inflammatory process. The study provides new insights into the immune system’s role in MDE, paving the ground for replication prospective studies targeting the development of diagnostic and prognostic tools and new therapeutic targets.

## Introduction

Depression is one of the most frequent mental disorders globally [1]; in many cases, it is recurrent and highly disabling, which makes it one of the leading causes of disability worldwide [2]. In addition to being associated with high levels of morbidity, it is estimated that 10% of depressed patients attempt suicide throughout the disease, inflating mortality rates [3]. Despite its substantial impact on morbidity and mortality, the underlying causes and mechanisms of depression remain poorly elucidated, hampering the development of more tailored therapeutic interventions to modify the disease state or progression.

The term “depression” typically refers to a Major Depressive Episode (MDE), according to the main international classifications. This category includes various psychiatric disorders, such as major depressive disorder (MDD), persistent depressive disorder (PDD), bipolar disorders (BD), adjustment disorders, or depressive disorder due to substance use or another medical condition [4]. Consequently, the clinical heterogeneity of depression is substantial, requiring at least five characteristics from a list of nine, with at least one of which must be low mood or anhedonia, to make the diagnosis [4]. This approach theoretically results in 227 potential combinations of criteria that qualify for an MDE diagnosis, even allowing for the possibility that two patients may receive the same diagnosis without sharing any symptoms [5]. In a study involving 1566 depressed individuals, researchers observed 170 unique symptom profiles [5]. Such clinical heterogeneity is reflected in the modest response exhibited by current treatments, which leaves a substantial subset of patients with treatment non-response. Unfortunately, we also lack biomarkers to discriminate across subgroups of MDEs or provide insights into their long-term evolution or treatment response. Immunology can significantly reduce diagnostic heterogeneity in patients with depression by providing additional insights into the underlying mechanisms of the disease.

Although the relationship between the immune system and depression is not novel, in recent years, growing evidence emphasized the crucial role of the immune system in the development and maintenance of depression [6]. It has been proposed that inflammation contributes to the clinical scenario and sickness context that lead to chronic maladaptive behavior [7–9]. In this sense, most studies have focused on humoral proinflammatory biomarkers, such as interleukin (IL)-6, tumor necrosis factor-alpha (TNFα) and C-reactive protein (CRP) [10–13]. Moreover, the most extensive meta-analysis up to date, analyzing a total of 107 studies reporting measurements of 5,166 patients with depression and 5,083 controls, found increases in the mean levels of CRP, IL-3, IL-6, IL-12, IL-18, soluble IL-2 receptor (sIL-2R) and TNFα in patients with depression [14]. Furthermore, an umbrella review conducted to evaluate non-genetic peripheral biomarkers in major mental disorders analyzed a dataset comprising 733,316 biomarker measurements of 162 biomarkers. The review revealed that only 42 biomarkers met the criteria for highly suggestive evidence. Among these, peripheral elevation of CRP was observed in both bipolar disorder (BD) and major depressive disorder (MDD), suggesting a potential association with peripheral inflammation. Additionally, increased sIL-2R levels were observed in MDD, indicating a possible contribution of immune-regulatory mechanisms to the pathophysiology of the disorder [15]. Despite the extensive research on humoral biomarkers, less exploration has been made regarding the involvement of innate and adaptive immune cells in depression [16, 17]. Of note, our research group has been at the forefront of this area, demonstrating significant alterations in the proportion and activation of the three subtypes of circulating monocytes in patients with severe MDD [18]. These observations have been replicated by others [19]. Furthermore, Lynall et al. [20], in a case–control study, proposed the existence of a peripheral cell-stratified subgroup termed “Inflamed depression”. This subgroup is differentiated by distinct myeloid- versus lymphoid-biased immune cell profiles, providing additional insights into the complex interplay between immune cells and depression [20]. A recent meta-analysis confirmed widespread alterations in circulating myeloid and lymphoid cells, consistent with dysfunction in both innate and adaptive immunity [21]. Introducing a biologically characterized phenotype of MDE into classification systems will hold substantial clinical relevance.

Inflammation is a biological phenomenon that can be thought as a response, process, or system state of any perturbations, including physiological and behavioral defenses to promote adaptation to environmental stressors [22, 23]. A deeper understanding of inflammation and its mediators should lead to advances in the therapeutics of mood disorders. In the current research landscape in immunology and depression, it is crucial to address a common limitation: many studies solely focus on soluble factors or cellular components of the proinflammatory response, potentially neglecting the broader picture. Therefore, the present study aims to comprehensively characterize and integrate various biochemical parameters, the pro- and anti-inflammatory humoral response, and the cellular compartment of the innate and adaptive immune response in patients with mood disorders experiencing an active major depressive episode (MDE), compared with those with a remitted MDE and healthy controls (HC). Our hypotheses are: (1) Patients with an active MDE will exhibit distinct immunological profiles compared to those with a remitted MDE and HC, (2) Specific biological markers identified through Boruta and Random Forest methods will accurately differentiate between MDE patients and HC, aiding in effective categorization and potentially serving as diagnostic or prognostic biomarkers and (3) Cluster analysis will reveal whether the immunological profiles observed in MDE patients indicate a singular immune system activation profile or suggest the presence of multiple distinct profiles, providing insights into the potential heterogeneity of immune system activation patterns in patients with MDE. By elucidating these immunological profiles and their associations with clinical characteristics, we aim to potentially identify distinct patient subgroups, paving the way for novel therapeutic strategies.

## Methods

### Study design

This multicenter case–control sex and age-matched study started recruiting participants in March 2019 and finished in December 2022. The patients were recruited from the *Hospital General de Agudos “Dr. Teodoro Álvarez”, Hospital General de Agudos “Dr. Enrique Tornú”, Hospital General de Agudos “Dr. Cosme Argerich”, Hospital General de Agudos “José María Ramos Mejía”*, and *Hospital Neuropsiquiátrico “Dr. Braulio A. Moyano”* in Buenos Aires. All these Hospitals serve a sizable urban catchment area in Buenos Aires and treat mainly low-income patients without insurance. The Institutional Review Board of each Hospital approved the study.

### Sample

Patients meeting the following criteria were included: (a) age between 18 and 65 years, (b) diagnosed with DSM-5 MDD or bipolar disorder (BP) in a current or remitted MDE (c) willing and able to sign a consent form to participate. Exclusion criteria were: (a) have a comorbid diagnosis of obsessive-compulsive disorder (OCD), psychotic disorders, or posttraumatic stress disorder (PTSD), (c) a diagnosis of borderline personality disorder (BPD), or (d) diagnosis of substance use disorder within the past 30 days.

Sex and age-matched healthy controls between 18 and 65 were recruited from the same community, ensuring a comparable sample. Exclusions for HC were: (a) having the diagnosis of any mental disorder, (b) having a diagnosis of substance use disorder within the past 30 days, (c) having a first-degree relative diagnosed with a mood disorder, d) not having the ability to sign a consent form.

Additional exclusion criteria for both the participants (MDE patients and HCs) were (a) the presence of a chronic or acute physical illness with an inflammatory component such as lupus erythematosus, rheumatoid arthritis, asthma, and celiac disease, among others. (b) receiving medication with anti-inflammatory or immunomodulatory properties, (c) getting infected with SARS-CoV-2 within the past 30 days before the evaluation, (d) having received the vaccine for SARS-CoV-2 or any other vaccine in the 30 days previous to the evaluation, (e) being pregnant, breastfeeding, having had an abortion or miscarriage during the previous 30 days to the evaluation.

### Measures

A trained interviewer gathered information regarding participant characteristics, including questions regarding clinical and demographic variables. The International Neuropsychiatric Interview (MINI), version 7.0.2 [24] was used for diagnostic purposes, and the 17-item Hamilton Depression Rating Scale (HDRS-17) [25] to establish the severity of the MDE.

Then, three groups of participants were defined: Group 1, “Patients with an active MDE”. The diagnosis of MDE as well as the type of mood disorder (MDD or BD), was determined by the MINI interview. Depression severity was established with the Hamilton Depression Rating Scale 17 (HDRS-17), and a score of >7 was used to define an active MDE. Group 2, “Patients with remitted MDE.” The diagnosis of a history of MDE and type of mood disorders was determined by MINI. Depression severity with HDRS-17 and a score of ≤7 was used to define a remitted MDE. Group 3, “Healthy Control” (HC) participants did not meet any diagnostic criteria by the MINI and scored ≤7 on the HDRS-17. The cutoff score of 7 on the HDRS-17 follows the recommendation by the NICE guidelines for depression [26].

Moreover, other questionnaires were used to control for other potential sources of variations in the inflammatory level beyond depression, the Columbia-Suicide Severity Rating Scale (C-SSRS) [27] to define if the patients had suicidal ideation or behavior, the Adverse Childhood Experiences (ACEs) questionnaire, the Brugha Stressful Life Events Scale [28], and the International Physical Activity Questionnaire (IPAQ) [29]. Weight and height were likewise recorded.

### Blood sample collection, processing, and biochemical analysis

Blood samples were drawn by venipuncture and collected into EDTA-coated tubes (BD, Vacutainer) in the morning before 10 AM on the same day as the psychiatric evaluations. Patients were not required to fast. A total of 20 mL of blood was obtained on the day of the clinical assessment. From these, 10 mL was used for routine biochemical laboratory tests, including the hemogram analysis, erythrocyte sedimentation rate (ESR), and high-sensitivity C-reactive protein (hs-CRP) measurements. The remaining blood sample was used for the direct Immunophenotyping staining, plasma separation, and peripheral blood mononuclear cells (PBMC) isolation as previously described [30].

### Immunophenotyping by direct blood staining

Three different antibody cocktails were used to determine the circulating monocyte subsets proportion, the activation markers on T cells, and the frequency of Tregs employing the appropriate combination of the following anti-human antibodies (BioLegend) **(1) Monocytes cocktail**: CD11b-Brilliant Violet 421™ (Cat # 101251, RRID: AB_2562904), HLA-DR-PE (Cat # 307606, RRID: AB_314684), CD86-biotin (Cat # 305404, RRID: AB_314524) plus DyLight™ 649-conjugated Streptavidin (Cat # 405224), CD14-PE/Cyanine7 (Cat # 325618, RRID: AB_830691), and CD16-fluorescein isothiocyanate (FITC) (Cat # 302005, RRID: AB_314205); **(2) T-cell cocktail**: CD3-PE/Cyanine7 (Cat # 300316, RRID: AB_314052), CD4-APC/Cyanine7(Cat # 317418, RRID: AB_571947), CD8-PE (Cat # 317418, RRID: AB_571947), CD69-PerCP/Cyanine5.5 (Cat # 310926, RRID: AB_2074956), CD44-BV421(Cat # 103040, RRID: AB_2616903), PD1-APC (Cat # 621610, RRID: AB_2832830) and LAG3-Alexa Fluor 488 (Cat # 369326, RRID: AB_2721362) and, **(3) Tregs**: CD3-PECy7(Cat # 317418, RRID: AB_571947), CD4-APCCy7 (Cat # 317418, RRID: AB_571947), CD25-Alexa Fluor 647 (Cat # 302618, RRID: AB_493045) (surface) and FOXP3-PE (Cat # 320108, RRID: AB_492986) (intracellular).

The direct staining in 100 µL of fresh anti-coagulated blood sample was standardized in our lab [31]. Briefly, for cell surface antigen staining, samples were incubated with the appropriate antibody cocktail on ice for 30 min in the dark and then fixed with 100 µL of Citofix Buffer (BD Bioscience) for an additional 20 min on ice. Then, cells were washed with PBS and centrifuged at 800 x *g* for 5 min. Next, to eliminate erythrocytes, the bottom of blood cells was incubated with 1 mL ACK Lysing Buffer (Thermofisher Scientific) for 10 min at 25 °C.

Only for Tregs, intracellular staining was performed after surface staining, and a specific kit (True-Nuclear™ Transcription Factor Buffer Set, Biolegend) was used. Briefly, 300 µL of 1x True Nuclear Fixation Buffer was added and incubated for 60 min at room temperature and in the dark. After that, cell permeabilization was performed by centrifuging the cells at 800  x g for 5 min with 200 µL of the True Nuclear 1x Perm Buffer, repeated twice. The FOXP3 antibody, diluted in True Nuclear 1x Perm Buffer, was added and incubated for 30 min in the dark at room temperature. Cells were maintained with 1x Perm Buffer, and finally, all three cocktails were washed with 1 mL PBS and analyzed by flow cytometry (BD Canto I) employing the FlowJo software.

The three monocyte subsets were defined by the expression of CD16 vs. CD14 as classical (CD16^neg^CD14^++^), non-classical (CD16^++^CD14^neg^), and intermediate (CD16^+^CD14^+^) as previously reported by our group [18]. In addition, the activation status of CD3^+^CD4^+^ lymphocytes was measured by the expression levels of the activation markers CD69 and CD44 and exhaustion markers PD1 and LAG3. Finally, the frequency of Tregs was determined by CD3^+^CD4^+^CD25^+^FOXP3^+^ cells.

### Plasma level of cytokines, chemokines, and neurotrophic factors determined by bead-based immunoassay

Plasma levels of cytokines, chemokines, and neurotrophic factors were measured using two LEGENDplex Panels (Biolegend) that allow the simultaneous quantification of several molecules in 50 μL of the plasma sample. LEGENDplex customized Human Inflammation Panel 1 was employed to measure (IL-1β, IFNγ, IL-17A, IL-33, CXCL8, IL-10, IL-12p70 and IL-23) and the LEGENDplex Human Neuroinflammation Panel 1 to measure (TGF-β, β-NGF, CX3CL1, BDNF, sTREM-2, IL-18, TNFα and CCL2).

All experiments were performed following the manufacturer’s instructions. The system is a bead-based multiplex assay panel using fluorescence-encoded beads, which can be read by flow cytometry and provide a standard curve to obtain concentrations of each cytokine based on the mean fluorescence intensity of the PE channel.

Additionally, IL-6 was determined by a high-sensitivity ELISA kit (Enzo Life Sciences) following the manufacturer’s instructions.

In our measurements, we have used commercially available standardized kits and considered the LOD and LOQ provided for each specific kit or reagent by the manufacturer (Supplementary Table S20). For each assay, we controlled the reagent lot and plate variations, by the inclusion of the standard curve provided by the manufacturer to ensure measurements were reliable. We have also added internal controls for each measurement of a previously determined sample.

### Analysis by flow cytometry

The samples were run in an external FACS core facility from the National System of Flow Cytometry, Argentina (FACS Canto I, Becton Dickinson). Data were analyzed by Flowjo software (Tree Star Inc). Bivariate dot plots with appropriate parameters were selected to define the gating strategy. The threshold for positivity was set using fluorescence minus one (FMO) for each marker.

### Data analysis

**Fig. 1:**
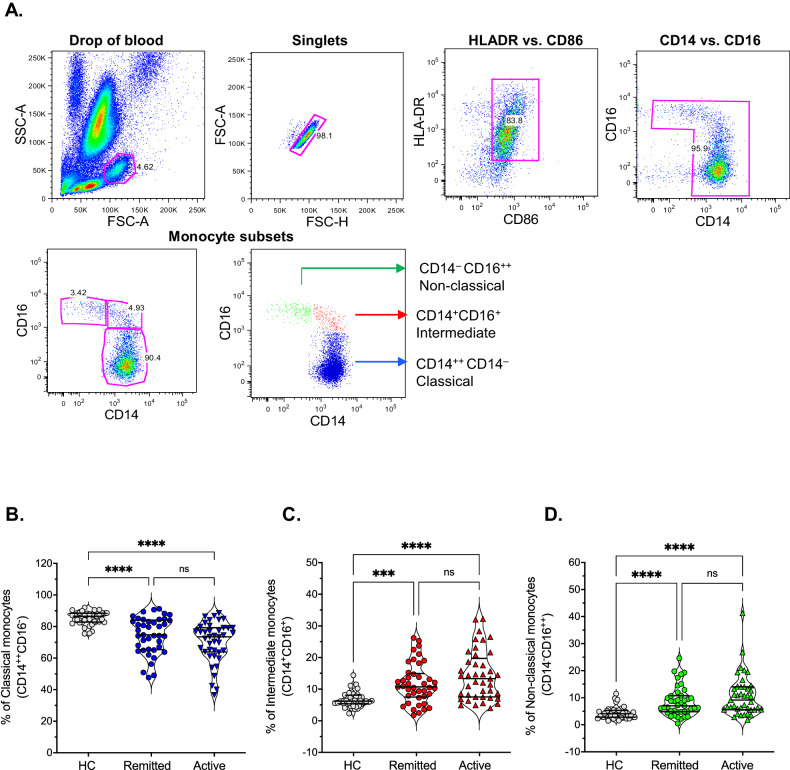
Sensitive screening of circulating monocytes subsets by direct blood staining in patients with MDE showed an altered proportion compared to control. **A** Representative dot plots, after fixation and RBCs lysis, show gating strategies based on forward scatter (FSC-A) versus side scatter (SCC-A) and then discrimination of singlets. After that, representative dot plots show the sequential gating based on the expression of HLA-DR versus CD86 first, and then, CD14 versus CD16. Finally, the 3 monocyte subsets are represented in blue for classical, in red for intermediate, and green for non-classical ones. **B**–**D** Independent data showing the percentage of classical (CD14^++^CD16^-^), intermediate (CD14^+^CD16^+^), and non-classical (CD14^−^CD16^++^) monocytes determined by flow cytometry. The median and interquartile range of each group is shown. Major depression episode (MDE), active vs remitted MDE, Healthy Control (HC). *Statistical differences among groups was calculated by Kruskal–Wallis rank sum test with Bonferroni adjustment for multiple comparisons*. ****P-value < 0.0001; ***P-value < 0.001; ns: not significant.

**Fig. 2:**
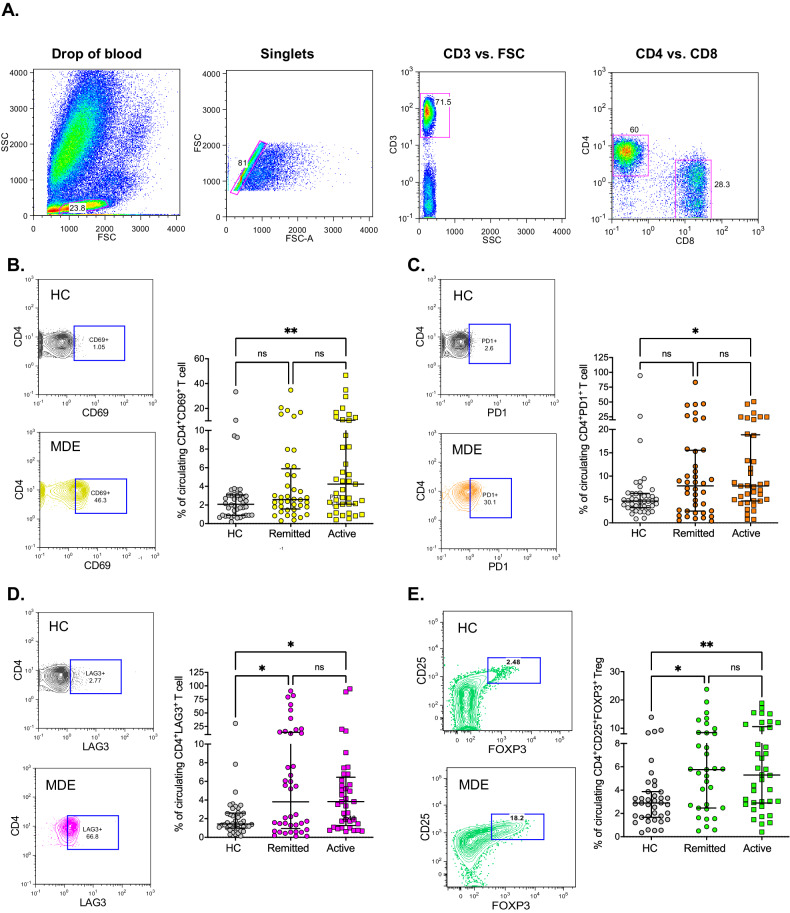
CD4 lymphocytes in patients with MDE showed increased activation and exhaustion markers with higher frequency of FOXP3 regulatory T cells. **A** Representative dot plots, after fixation and RBCs lysis, showing gating strategies based on forward scatter (FSC) versus side scatter (SCC) and then discrimination of singlets. After that, representative dot plots show the sequential gating based on the expression of CD3 versus FSC first, and then, CD4 versus CD8. **B**–**D** Representative dot plots and the independent data showing the percentage of CD69^+^, PD1^+^ and, LAG3^+^ on CD4^+^ lymphocytes and, **E** the representative dot plot and percentage of CD4^+^CD25^+^FOXP3^+^ regulatory T cells, determined by flow cytometry. The median and interquartile range of each group is shown. Major Depression Episode (MDE), active vs remitted MDE, Healthy Control (HC). *Statistical differences among groups were calculated by Kruskal–Wallis rank sum test with Bonferroni adjustment for multiple comparisons*.**P-value < 0.01; *P-value < 0.05; ns: not significant.

**Fig. 3:**
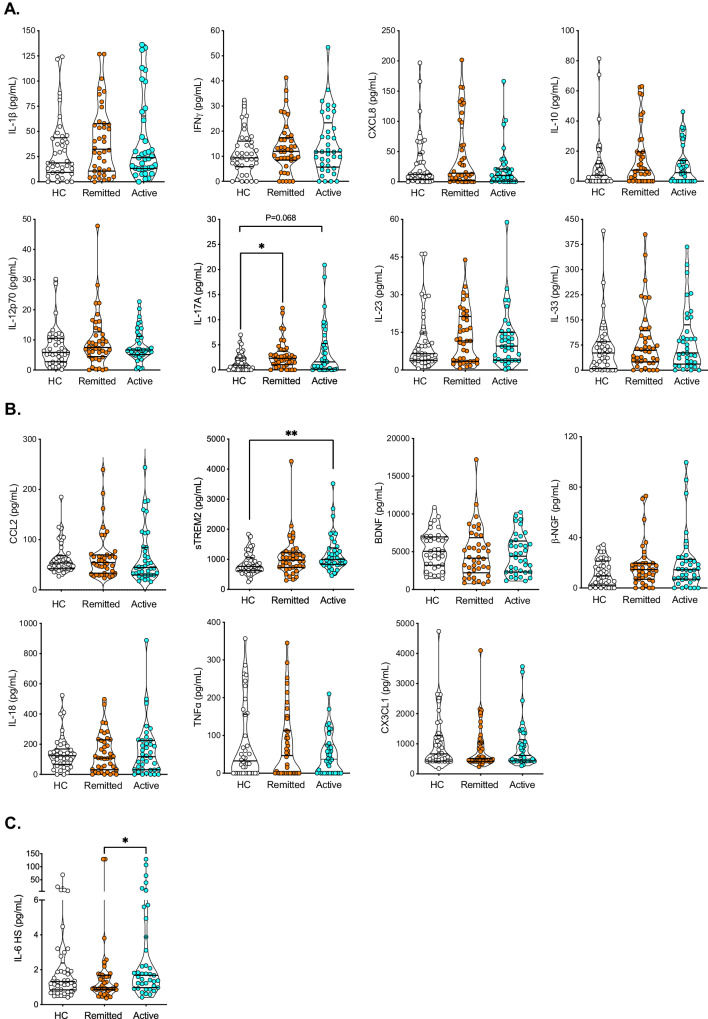
Inflammatory and Neuroinflammation panel measured in plasma of patients with MDE showed increased levels of sTREM2, IL-17A, and IL-6. **A**, **B** Cytokines, chemokines, and neurotrophic factors, were assessed employing a customized LEGENDPlex system for a human inflammatory panel 1 (**A**), and a neuroinflammation panel (**B**) determined by flow cytometry. Each panel allow the simultaneous quantification of all assessed molecules in 50 µL of the sample. The concentrations of each cytokine are determined by a standard curve provided by the kit, see Supplemental Figs. S1A and S1B. **C** IL-6 was determined by high-sensitivity (HS) ELISA kit. Independent data for each molecule is graphed and the median of each group is shown. Human Inflammatory Panel 1 includes: IL-1β, IFNγ, IL-17A, IL-33, CXCL8, IL-10, IL-12p70 and IL-23, and the Human Neuroinflammation Panel includes: β-NGF, CX3CL1, BDNF, sTREM2, IL-18, TNFα and CCL2. Major Depression Episode (MDE), active vs remitted MDE, Healthy Control (HC). *Statistical differences among groups was calculated by Kruskal–Wallis rank sum test with Bonferroni adjustment for multiple comparisons*. **P-value < 0.01; *P-value < 0.05.

All statistical analyses were performed using RStudio 2022.02.1 + 461 [32]. Descriptive statistics were used to summarize participants’ characteristics. Categorical variables were reported as absolute and relative frequencies (%), while quantitative variables were reported as means and standard deviations (SDs) for normally distributed variables or as the median and interquartile range (IQR) for non-normally distributed ones. The Shapiro-Wilk test was used to assess the normality of each quantitative variable. The graphics and statistical comparison of Figs. 1, 2, and 3 were performed using GraphPad Prism software.

To test our first hypothesis, we compared the immunological profiles among participants in the three groups based on the variable type. Specifically, categorical variables were compared using Pearson’s Chi-squared or Fisher’s exact test, normally distributed quantitative variables were compared using ANOVA, and non-normally distributed quantitative variables were compared using the Kruskal–Wallis rank sum test. Pairwise comparisons were performed for variables with significant between-group differences. The Specific post-hoc test and multiple comparison test employed have been detailed in the figure legends. Adjustment of p-values was performed for multiple comparisons among groups for those variables with a statistically significant difference in the bivariate analysis. In the case of means/median comparison tests for the different variables, the reported p-values were not adjusted. Adittionally, a secondary analysis was conducted to compare the inflammatory and immunological profiles of patients with unipolar depression (UD) and bipolar depression (BD). A significance level of *p* < 0.05 was considered statistically significant.

For subsequent analyses, missing data were imputed using the k-nearest neighbors method with a value of *k* = 5, using the kNN option in the VIM package [33].

The sample size of the three groups was calculated considering as objective the identification of significant differences in the quantitative variables by means of the ANOVA test. For a significance level of 5%, a power of 80%, and an effect size *f* = 0.30 (a medium effect), a total of 111 individuals was required, 37 patients per group. We have computed a needed sample size for one-way ANOVA using G*Power 3.1.9.4 software.

The correlation between variables was evaluated using the Spearman correlation coefficient.

To test our second hypothesis and select the most important variables for classifying individuals as patients or healthy controls, we employed Random Forest, in combination with the Boruta algorithm [34]. The dataset was split into a training set comprising 70% of the observations and a test set comprising the remaining 30% while maintaining the proportionality of groups in the original data. Boruta was applied to the training data, and then a Random Forest model with the selected variables was fitted to the test data to evaluate the classification performance of the obtained models.

To test our third hypothesis and determine if the immunological profiles in MDE patients indicate a singular immune system activation profile or suggest multiple distinct profiles, a clustering analysis was conducted, considering both active and remitted cases. Initially, a principal component analysis was applied to reduce the dimensionality of the data. The permutation-based test was used to determine the number of components to retain, employing the factoextra [35] and PCAtest packages. Subsequently, a hierarchical clustering analysis consolidated by k-means clustering was conducted based on the retained factors from the previous analysis. The suggested partition was determined based on the relative gain of inertia using the HCPC option in the FactoMineR package [36].

## Results

### Sociodemographic and clinical characteristics of the participants

The sociodemographic and clinical characteristics of the participants are summarized in Table 1. We recruited 121 participants: 39 patients with an active (current) MDE (32.2%), 40 with a remitted MDE (33.1%), and 42 HC subjects (34.7%). The three groups did not differ in sex and age, showing an appropriate matching. As anticipated, patients showed higher levels of unemployment and lower educational level compared to HC. On the other hand, as expected, the patients presented higher scores on the HAMD-17, higher levels of suicidal risk, and more than 85% were undergoing psychopharmacological treatment. Also, patients showed higher levels of adverse childhood events and stressful events. Additional clinical characteristics of the patients are described in Supplementary Table S1.

**Table 1:** Sociodemographic and clinical characteristics of participants.

Variable	Active MDE	Remitted MDE	Healthy Control	*p*-value
*n*	39	40	42	
Age (median [IQR])	43.00 [34.50, 50.50]	41.00 [29.50, 52.25]	39.00 [30.25, 49.00]	0.654
Gender = Male (%)	10 (25.6)	14 (35.0)	14 (33.3)	0.633
Civil status (%)				0.189
Married/living with a partner	11 (28.2)	5 (12.5)	13 (31.0)	
Separated/divorced/widower	8 (20.5)	11 (27.5)	5 (11.9)	
Single	20 (51.3)	24 (60.0)	24 (57.1)	
Scholarship (%)				NA
None	0 (0.0)	0 (0.0)	0 (0.0)	
Incomplete primary	0 (0.0)	0 (0.0)	0 (0.0)	
Complete primary	3 (7.7)	2 (5.0)	0 (0.0)	
Incomplete high school	11 (28.2)	4 (10.0)	1 (2.4)	
Complete high school	6 (15.4)	5 (12.5)	2 (4.8)	
Incomplete college	10 (25.6)	18 (45.0)	16 (38.1)	
Complete college	9 (23.1)	11 (27.5)	23 (54.8)	
Employment status = unemployed/retired (%)	20 (51.3)	16 (40.0)	5 (11.9)	**0.001**
Diagnostic summary (%)				NA
Bipolar disorder I	6 (15.4)	21 (52.5)	0 (0.0)	
Bipolar disorder II	10 (25.6)	2 (5.0)	0 (0.0)	
Major depressive disorder	23 (59.0)	16 (40.0)	0 (0.0)	
Non-specific Bipolar disorder	0 (0.0)	1 (2.5)	0 (0.0)	
None depressive disorder	0 (0.0)	0 (0.0)	42 (100.0)	
HAM-D Total score (median [IQR])	14.00 [11.00, 18.00]	3.50 [1.00, 5.00]	0.00 [0.00, 1.00]	**<0.001**
On psychopharmacological treatment = Yes (%)	31 (81.6)	38 (95.0)	0 (0.0)	**<0.001**
Total number of ACEs (median [IQR])	4.00 [2.00, 6.00]	3.50 [2.00, 6.00]	0.00 [0.00, 1.75]	**<0.001**
IPAQ (%)				0.609
Low	23 (59.0)	18 (45.0)	19 (45.2)	
Median	8 (20.5)	9 (22.5)	12 (28.6)	
High	8 (20.5)	13 (32.5)	11 (26.2)	
Suicide ideation month = Yes (%)	19 (55.9)	2 (8.7)	0 (0.0)	**<0.001**
Suicide Behavior month = Yes (%)	1 (2.6)	1 (2.5)	0 (0.0)	0.543
Suicide ideation life = Yes (%)	30 (85.7)	30 (100.0)	0 (0.0)	**<0.001**
Suicide Behavior life = Yes (%)	13 (33.3)	16 (40.0)	0 (0.0)	**<0.001**
Number of stressful life events (median [IQR])	10.00 [7.00, 14.00]	10.00 [7.75, 14.00]	8.50 [6.00, 12.00]	0.156
Stressful life events total score (median [IQR])	307.00 [206.50, 444.00]	298.00 [197.50, 426.25]	187.50 [139.25, 316.50]	**0.001**
Weight (median [IQR])	70.00 [60.05, 79.00]	66.80 [60.00, 84.25]	70.00 [60.75, 78.50]	0.999
Height (mean (SD))	165.38 (8.92)	164.93 (8.59)	167.76 (8.73)	0.291
BMI (median [IQR])	24.40 [22.55, 30.80]	25.70 [22.75, 30.10]	24.25 [22.00, 27.67]	0.422
Smokers = yes (%)	20 (51.2)	17 (42.5)	5 (11.9)	**<0.001**
Amount of cigarettes per day (median [IQR])	0.00 [0.00, 19.00]	0.00 [0.00, 6.50]	0.00 [0.00, 0.00]	**0.001**
Alcohol use = yes (%)	17 (43.6)	11 (27.5)	31 (75.6)	**<0.001**

*MDE* major depressive episode, *HAM-D* Hamilton Rating Scale for Depression, *ACEs* adverse childhood experiences questionnaire, *IPAQ* International Physical Activity Questionnaire, *BMI* body mass index.

*p*-values were calculated using Pearson’s Chi-squared or Fisher’s exact test (Categorical variables), ANOVA (normally distributed quantitative variables), and Kruskal–Wallis rank sum test (non-normally distributed quantitative variables).

Bold values are used to highlight statistical significance.

### The inflammatory status of MDE patients is characterized by monocytosis and an altered proportion of monocyte subsets

The routine biochemical laboratory tests, using an automated cell counter and analyzer, are summarized in Table 2. Significant differences were observed when comparing HC and MDE patients with active disease. These differences were observed in the median percentage of monocytes (*p* = 0.011) and their absolute count (*p* = 0.001) and for the mean percentage of lymphocytes (*p* = 0.004), even though its absolute number did not change, among groups.

**Table 2:** Comparison of the biochemical parameters among participants.

Variable	Active MDE	Remitted MDE	Healthy control	*p*-value
Hematocrit (median [IQR])	41.20 [39.18, 43.55]	41.65 [39.42, 44.95]	42.50 [40.90, 44.30]	0.631
Hemoglobin (median [IQR])	13.30 [12.80, 14.40]	13.80 [12.93, 14.78]	14.10 [13.40, 14.70]	0.269
Erythrocytes (mean (SD))	4.73 (0.52)	4.71 (0.53)	4.85 (0.40)	0.318
MCV (median [IQR])	88.73 [85.20, 91.21]	89.00 [85.74, 91.40]	86.47 [84.06, 89.54]	**0.047**
MCH (median [IQR])	29.00 [28.08, 30.02]	29.00 [27.88, 30.00]	28.79 [27.86, 29.71]	0.653
MCHC (median [IQR])	32.80 [32.50, 33.45]	32.90 [32.38, 33.83]	33.33 [32.51, 33.96]	0.137
Leukocytes (median [IQR])	7.40 [6.10, 9.80]	6.56 [5.20, 8.00]	6.60 [5.70, 7.50]	0.138
Segmented neutrophils Percentage (median [IQR])	58.00 [52.00, 66.00]	60.00 [50.00, 64.60]	55.00 [50.00, 60.00]	0.154
Segmented neutrophils absolute count (median [IQR])	4.02 [3.35, 5.68]	3.69 [2.70, 4.96]	3.59 [3.02, 4.42]	0.134
Lymphocytes percentage (mean (SD))	32.38 (7.64)	35.62 (11.36)	38.16 (6.83)	**0.004**
Lymphocytes absolute count (median [IQR])	2.44 [1.86, 2.98]	2.09 [1.87, 2.42]	2.52 [2.15, 2.77]	0.059
Monocytes percentage (median [IQR])	5.50 [3.00, 8.00]	4.50 [3.00, 7.75]	3.00 [2.00, 5.00]	**0.011**
Monocytes absolute count (median [IQR])	0.39 [0.27, 0.63]	0.30 [0.19, 0.52]	0.22 [0.14, 0.30]	**0.001**
Eosinophils percentage (median [IQR])	2.00 [2.00, 2.00]	2.00 [1.15, 2.00]	2.00 [1.00, 3.00]	0.756
Eosinophils absolute count (median [IQR])	0.15 [0.11, 0.20]	0.13 [0.09, 0.20]	0.13 [0.10, 0.20]	0.448
Basophils percentage (median [IQR])	0.00 [0.00, 0.30]	0.00 [0.00, 0.00]	0.00 [0.00, 0.00]	0.137
Basophils absolute count (median [IQR])	0.00 [0.00, 0.02]	0.00 [0.00, 0.00]	0.00 [0.00, 0.00]	0.137
ESR (median [IQR])	15.00 [10.00, 21.00]	10.00 [7.00, 13.25]	10.00 [6.25, 12.00]	**0.003**
Urea (median [IQR])	29.00 [22.75, 34.25]	29.50 [25.25, 36.00]	29.00 [23.00, 36.00]	0.846
Creatinine (median [IQR])	7.38 [0.94, 9.30]	7.70 [0.95, 10.10]	8.10 [7.22, 10.00]	0.166
GOT (median [IQR])	23.50 [15.25, 30.75]	23.00 [19.00, 31.00]	25.00 [18.00, 30.00]	0.903
GPT (median [IQR])	20.50 [13.25, 33.00]	29.00 [20.00, 34.00]	24.00 [18.00, 31.00]	0.276
Alkaline phosphatase (median [IQR])	125.00 [81.00, 189.00]	159.50 [121.75, 202.00]	155.00 [118.00, 179.00]	0.365
Total bilirubin (median [IQR])	0.48 [0.38, 0.70]	0.48 [0.34, 0.64]	0.66 [0.50, 0.86]	**0.013**
Sodium (mean (SD))	137.85 (2.88)	138.11 (4.16)	138.37 (3.21)	0.757
Potassium (median [IQR])	4.30 [4.00, 4.60]	4.30 [4.00, 4.62]	4.30 [4.00, 4.50]	0.729
Chloride (median [IQR])	99.00 [90.00, 100.00]	99.00 [90.00, 101.55]	93.00 [89.00, 99.00]	0.113
hs-CRP (median [IQR])	2.81 [0.52, 9.15]	1.19 [0.56, 3.59]	0.55 [0.20, 1.92]	**0.002**

*MDE* major depressive episode, *MCV* mean corpuscular volume, *MCH* mean corpuscular hemoglobin, *MCHC* mean corpuscular hemoglobin concentration, *ESR* erythrocyte sedimentation rate, *GOT* glutamic-oxaloacetic transaminase, *GPT* glutamic pyruvic transaminase, *hs-CRP* high-sensitivity C-reactive Protein.

*Units of measurement:* Hematocrit *(%)*, Hemoglobin *(g/dL)*, Erythrocytes *(10*^*12*^ *cells/L), MCV (femtolitres), MCH (picograms/cell), MCHC (g/dL)*, and Leukocytes, segmented neutrophils, lymphocytes, monocytes, eosinophils, and basophils are expressed as *(10*^*9*^ *cells/L). ESR (mm/hour)*, urea *(mg/dL)*, creatinine *(mg/dL), GOT (IU/L), GPT (IU/L)*, Alkaline phosphatase *(IU/L)*, and total bilirubin *(mg/dL)*. sodium, potassium, and chloride are expressed as *(mmol/L)*, and *hs-CRP (mg/L)*.

*p*-values were calculated using ANOVA (normally distributed quantitative variables) and Kruskal–Wallis rank sum test (non-normally distributed quantitative variables).

Bold values are used to highlight statistical significance.

Moreover, the median percentage of monocytes (*p* = 0.016) and their absolute number (*p* < 0.001) were increased in patients with active MDE compared to HC. These results indicate that the main hematopoietic response is coming from monocytosis promotion. Additionally, a reduced percentage of lymphocytes (*p* = 0.0024) was also found in patients with active MDE compared to HC (Supplementary Fig. S1A). No significant changes were observed in other cellular compartments (neutrophils, eosinophils, basophils, including erythrocytes).

Another two peripheral blood inflammatory indicators are the hs-CRP and the ESR, and a chronic low-grade systemic inflammation could be reflected by a small but significant increment of their values. In this sense, we observed a significant difference in the concentration of hs-CRP (*p* = 0.002) and ESR value (*p* = 0.003), among groups, see Table 2. Furthermore, we detected an increase in the concentration of hs-CRP in active MDE and remitted MDE compared to HC (*p* = 0.004 and 0.021, respectively) and ERS value in active MDE compared to remitted MDE and HC (*p* = 0.028 and 0.003, respectively), see Supplementary Fig. S1A.

Considering the monocytosis and the increment in the systemic proinflammatory parameters hs-CRP and ESR in patients with MDE, we also evaluated changes in the proportion of the three subtypes of circulating monocytes, classical (CD14^++^CD16^-^), intermediate (CD14^+^CD16^+^), and non-classical (CD14^-^CD16^++^) by flow cytometry, as another proinflammatory hallmark. As we previously reported using peripheral blood mononuclear cells (PBMCs) [18], here we also found, by direct staining on fresh peripheral blood, a higher proportion of non-classical and intermediate monocytes in concordance with a reduced percentage of classical monocytes in both active and remitted patients with MDE vs. HC, see Fig. 1A–D.

### Increased activation and exhausted phenotype in CD4 lymphocytes of patients with MDE is associated with a higher frequency of FOXP3 regulatory T cells

Even though we observed a reduced percentage of total lymphocytes in the hemogram, no difference was found in the absolute number (Table 2). Neither the percentage of CD4 T cells nor the ratio of CD4/CD8 T cells measured by flow cytometry showed significant differences between patients with MDE and HC (Supplementary Table S2). Nonetheless, we observed a significant increment in the median percentages of activation markers CD69^+^ (*p* = 0.007), and exhaustion markers PD1^+^ (*p* = 0.013) and LAG3^+^ (*p* = 0.014), on CD4^+^ T lymphocytes in patients with active MDE compared to HC, see Fig. 2A–D. We did not observe significant changes in the CD44 activation marker (Supplementary Table S2).

Even though there is no consensus regarding the reduction or increase number of regulatory T cells (Tregs) in depression, we found here a significant increase in the frequency of CD4^+^CD25^+^FOXP3^+^ Tregs in patients with active (*p* = 0.003) as well as remitted MDE (*p* = 0.015) compared with HC, see Fig. 2E. No differences were observed between active vs. remitted MDE.

### Inflammatory and Neuroinflammation panels showed increased levels of sTREM2, IL-17A, and IL-6 in MDE patients

The LEGENDPlex system was used to assess sixteen molecules, including cytokines, chemokines, and neurotrophic factors, in the plasma of patients with MDE. The level of each molecule was quantified based on the standard curve (Supplementary Fig. S1A, S1B, and Table S20). This assessment utilized a customized human inflammatory panel (Fig. 3A) along with a human neuroinflammation panel (Fig. 3B). Interestingly, we have found a robust and significantly higher level of sTREM2, a biomarker of microglia activation, in patients with active MDE compared with HC (*p* = 0.0089), see Fig. 3B. We have also observed a significant higher level of IL-17A in patients with remitted MDE compared with HC (*p* = 0.0147), see Fig. 3A, and a trend in the median value comparing active MDE patients vs HC (*p* = 0.068). Although with no statistical differences, some classical cytokines from the innate and adaptive immune response (IL-1β, IL-12, IFNγ, and IL-10), showed an increased concentration trend, see Fig. 3A.

A strong positive correlation was observed between IL-10 and IL-12 (*r* = 0.781), IL-33 and IL-10 (*r* = 0.726), as well as IL-1β and IFNγ (*r* = 0.704), based on a correlation analysis using the Spearman test. For detailed correlation results, please refer to Supplementary Fig. S2 and Supplementary Table S3.

Considering that the IL-6 measurement was under the low range of detection of the legendPlex system, we have measured this cytokine in the plasma of HC and patients with MDE employing a highly sensitive ELISA kit (Enzo Life Sciences, Supplementary Table S20). We found that patients with active MDE show higher levels than the remitted MDE group (*p* = 0.0278) (Fig. 3C).

The only significant difference in the immunological and inflammatory profile between UD and BD was observed in the higher levels of CX3CL1 in patients with UD (*p* = 0.022, Supplementary Table 12).

### Boruta and Random Forest validate the discriminatory power of biological markers in patients with MDE

The Boruta selection algorithm was used to find the most important markers to discriminate individuals between MDE patients and HC, as well as active MDE, remitted MDE, and HC.

**Fig. 4:**
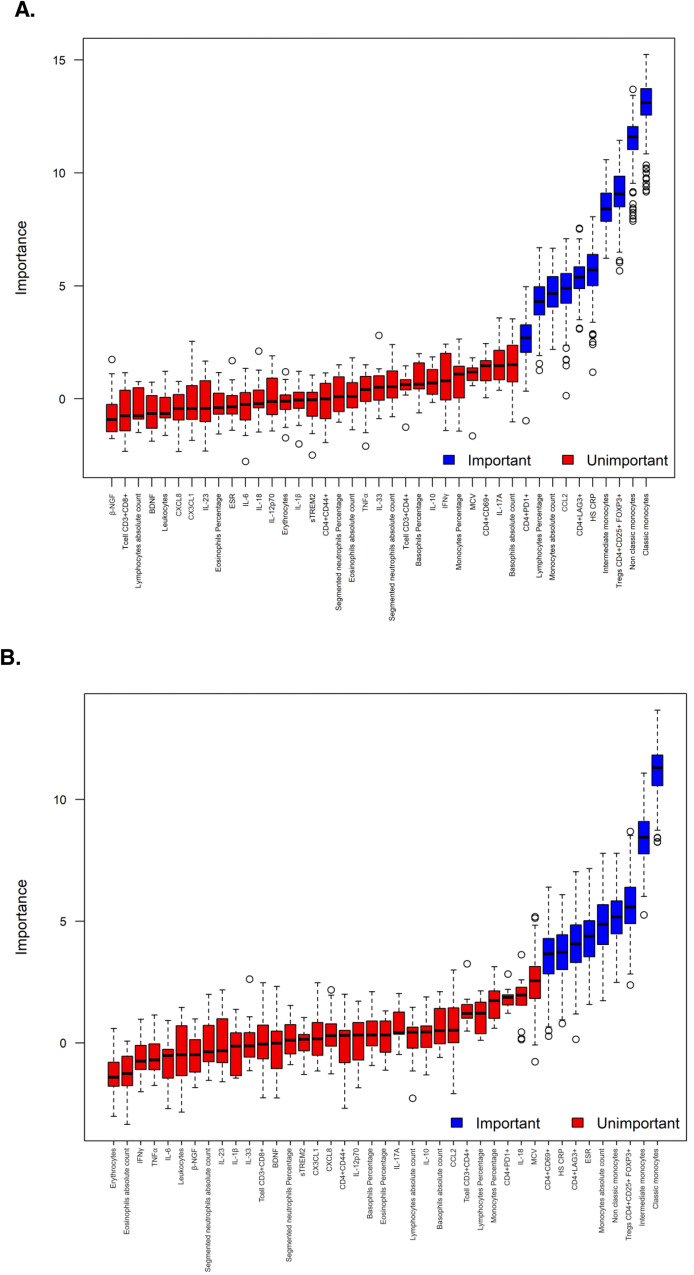
Boruta and Random Forest validate the discriminatory power of biological markers in patients with MDE. To select the most important variables for classifying individuals, Random Forest, in combination with the Boruta algorithm, was employed. **A** The first training set was performed with the 70% (*N* = 84) of the pool data discriminated by patients with MDE or HC. From this analysis, the most discriminatory variables were: lymphocyte percentage, monocytes absolute count, classical monocytes, non-classical monocytes, intermediate monocytes, hs-CRP, CCL2, CD4^+^PD1^+^, CD4^+^LAG3^+^ and CD4^+^CD25^+^ FOXP3^+^ Treg. **B** Secondly, the training model was applied to discriminating patients with active MDE, remitted, or HC. From this analysis, the most discriminatory variables were: classical monocytes, non-classical monocytes, intermediate monocytes, monocytes absolute count, ESR, hs-CRP, CD4^+^CD69^+^, CD4^+^LAG3^+^ and CD4^+^CD25^+^ FOXP3^+^ Treg.

First, the model was applied to discriminate between patients with MDE vs. HC. From the training set (*n* = 84), the variables lymphocytes percentage, monocytes absolute count, classical monocytes, non-classical monocytes, intermediate monocytes, hs-CRP, CCL2, CD4^+^PD1^+^, CD4^+^LAG3^+^ and CD4^+^CD25^+^ FOXP3^+^ Tregs were selected (Fig. 4A). These markers were used in a Random Forest model to classify a separate test dataset, achieving an overall classification accuracy of 83.8%. Twenty-one over twenty-four (21/24) patients with MDE were correctly classified (87.5%) and 10/13 HC (76.9%).

Afterwards, the model was applied to be able to discriminate among patients with active MDE, remitted MDE, and HC. Similarly, the Boruta selection algorithm identified markers that were important for discrimination: classical monocytes, non-classical monocytes, intermediate monocytes, monocytes absolute count, ESR, hs-CRP, CD4^+^CD69^+^, CD4^+^LAG3^+^ and CD4^+^CD25^+^FOXP3^+^ Tregs, (Fig. 4B). Random Forest model was then trained using these selected variables and applied to classify the test dataset, achieving an overall classification accuracy of 70%. Here, 7/12 patients with active MDE (58.3%), 8/12 remitted MDE (66.7%), and 10/13 HC (84.6%) were correctly classified.

### Clustering analysis indicates different inflammatory segregation among patients with MDE

**Fig. 5:**
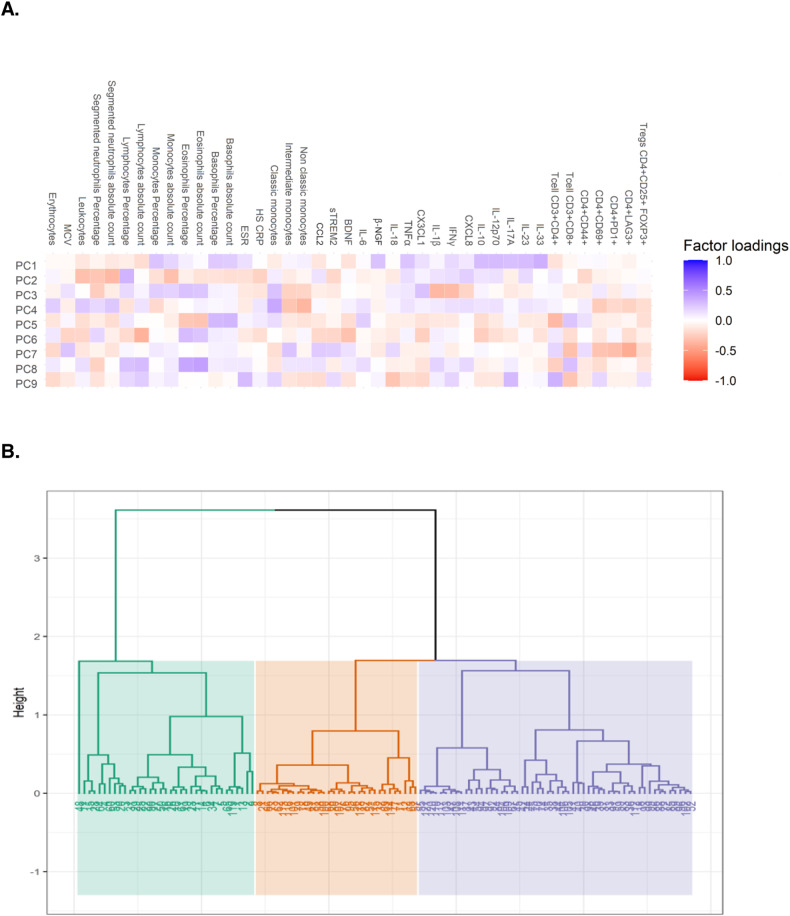
Clustering analysis shows different inflammatory groups within patients with MDE. A clustering analysis was performed considering both active and remitted cases. **A** Initially, a principal component analysis was applied to reduce the dimensionality of the data and work with the most significant contributors. **B** The hierarchical clustering analysis revealed 3 main segregated groups based on the 9 selected principal components obtained. All clinical, biochemical, and immunological characteristics of each cluster are summarized in Tables 3 and 4 and Supplementary Tables S4 and S5.

Principal component analysis (PCA) was applied to reduce the dimensionality of the data, yielding nine principal components, which together accounted for 62.5% of the total variance-covariance. This first procedure facilitates the subsequent cluster analysis since it will be carried out on a reduced number of variables (the selected principal components) instead of all the variables in the dataset. The first principal component (PC1, 14.2% total variance-covariance) was most strongly weighted on IL-17A, IL-23, IL-10, IL-12p70, IL-33, TNFα, β-NGF, the absolute count, and the percentage of basophils and the percentage of monocytes. The second principal component (PC2, 10.4% total variance-covariance) was most weighted on the absolute count and the percentage of segmented neutrophils, the absolute count of monocytes and leukocytes, the percentage of lymphocytes, HS-CRP, and CXCL8. The factor loadings of the variables in the nine principal components are represented in Fig. 5A.

The Clustering analysis was used to detect different inflammatory segregation among MDE patients. The suggested partition was determined based on the relative gain of inertia, which provides a firm criterion to determine the number of subgroups to consider. Three distinct clusters were identified, as revealed by the dendrogram graphic (Fig. 5B).

Comparing the clinical and sociodemographic characteristics among the three clusters we observed a similar distribution of the active or remitted condition of MDE with no significant statistical differences (Pearson’s Chi-squared, *p* = 0.623), neither on the severity of depressive symptoms considering HAMD-17 scale (Kruskal–Wallis rank sum test, *p* = 0.398). Nonetheless, cluster 1 and 3 showed median scores higher than 7 (HAMD-17 = 9.5 and 8, respectively) compared with cluster 2 (HAMD-17 = 6), see Supplementary Table S4. A significant difference was observed in the percentage of subjects receiving pharmacological treatment among clusters. In Cluster 1, 95.3% of the cases were under psychopharmacological treatment, as well as 95.0% in Cluster 2, in contrast, only 60% of individuals in Cluster 3 were receiving treatment (Fisher’s exact test, *p* = 0.001). The association between IPAQ and the assigned cluster was significant (Pearson’s Chi-squared, *p* = 0.039). In cluster 1, 29.5% of individuals had a high level on this scale, as did 40.0% of subjects in cluster 2. However, no cases with that level of IPAQ were observed in cluster 3.

The biochemical parameters analysis across these three clusters shows again that clusters 1 and 3 displayed higher median values of absolute leukocyte number. In concordance, cluster 1 showed the highest value of lymphocyte number, and cluster 3 the highest percentage and absolute number of monocytes (Table 3). Furthermore, cluster 3 also exhibited the highest medians for basophil percentage and absolute count.

**Table 3:** Comparison of biochemical parameters among clusters.

Variable	1	2	3	*p*-value
Erythrocytes (mean (SD))	4.73 (0.55)	4.72 (0.49)	4.69 (0.51)	0.981
MCV (median [IQR])	89.86 [87.00, 93.00]	88.44 [86.23, 90.01]	87.00 [84.50, 90.15]	0.177
Leukocytes (median [IQR])	7.68 [6.18, 9.83]	5.40 [5.00, 7.45]	6.56 [5.88, 7.30]	**0.021**
Segmented neutrophils percentage (mean (SD))	58.91 (11.16)	56.21 (9.70)	57.86 (6.94)	0.642
Segmented neutrophils absolute count (median [IQR])	4.46 [3.29, 6.84]	3.16 [2.50, 4.73]	3.84 [3.28, 4.72]	0.074
Lymphocytes percentage (median [IQR])	32.65 [25.00, 41.25]	38.00 [31.50, 43.00]	29.80 [25.50, 33.90]	0.073
Lymphocytes absolute count (median [IQR])	2.58 [2.07, 2.97]	2.09 [1.84, 2.27]	1.86 [1.68, 2.13]	**0.004**
Monocytes percentage (median [IQR])	4.00 [3.00, 8.00]	4.00 [3.00, 5.00]	8.60 [7.10, 9.95]	**<0.001**
Monocytes absolute count (median [IQR])	0.38 [0.21, 0.58]	0.21 [0.19, 0.33]	0.52 [0.46, 0.67]	**<0.001**
Eosinophils percentage (median [IQR])	2.00 [1.00, 2.00]	2.00 [2.00, 2.00]	2.00 [1.65, 4.45]	0.228
Eosinophils absolute count (median [IQR])	0.15 [0.09, 0.20]	0.11 [0.10, 0.18]	0.14 [0.11, 0.28]	0.726
Basophils percentage (median [IQR])	0.00 [0.00, 0.00]	0.00 [0.00, 0.00]	1.00 [0.55, 1.00]	**<0.001**
Basophils absolute count (median [IQR])	0.00 [0.00, 0.00]	0.00 [0.00, 0.00]	0.06 [0.03, 0.07]	**<0.001**
ESR (median [IQR])	10.00 [7.00, 15.00]	14.00 [9.00, 18.00]	16.00 [5.00, 26.25]	0.376
hs-CRP (median [IQR])	2.10 [0.51, 7.95]	1.49 [0.91, 3.67]	2.03 [0.59, 3.62]	0.952

*MCV* mean corpuscular volume, *ESR* erythrocyte sedimentation rate, *hs-CRP* high-sensitivity C-reactive protein.

*Units of measurement:* Erythrocytes *(10*^*12*^ *cells/L), MCV (femtolitres)*. Leukocytes, Segmented neutrophils, Lymphocytes, Monocytes, Eosinophils, and Basophils are expressed as *(10*^*9*^ *cells/L). ESR (mm/hour)*, and *hs-CRP (mg/L)*.

*p*-values were calculated using ANOVA (normally distributed quantitative variables) and the Kruskal–Wallis rank sum test (non-normally distributed quantitative variables).

Bold values are used to highlight statistical significance.

The proportion of the three monocyte subtypes is altered in patients with MDE, but the segregation in these three clusters did not show significant differences (Supplementary Table S5). Interestingly, the lowest percentage of CD3^+^CD4^+^ T cells, determined by flow cytometry, was observed in cluster 3, in concordance with the biochemical laboratory analysis. In the same sense, clusters 1 and 3 showed an increased median value of exhaustion CD4^+^LAG3^+^ marker compared to cluster 2 (Supplementary Table S5).

Considering cytokines, chemokines, and neurotrophic factors, the cluster segregation denoted that cluster 3 is characterized by a clear proinflammatory profile with high levels of TNFα, CX3CL1, IL-12p70, IL-17A, IL-23, and IL-33, associated with a high level of CXCL8 and IL-10, as well as increased medians for β-NGF and the lowest level for BDNF (Table 4). Cluster 2 is mainly characterized by the highest level of CCL2, IL-1β, IFNγ, IL-23, and CXCL8. Cluster 1 displayed the lowest median level of most cytokines (Table 4).

**Table 4:** Inflammatory and neuroinflammation panel of cytokines among clusters.

Variable	1	2	3	*p*-value
CCL2 (median [IQR])	52.53 [33.01, 78.63]	64.17 [46.09, 111.27]	29.73 [26.18, 46.45]	**0.002**
sTREM2 (median [IQR])	976.33 [779.43, 1300.05]	1044.76 [748.32, 1263.49]	931.84 [736.20, 1170.49]	0.847
BDNF (median [IQR])	5120.64 [2920.72, 7086.43]	4854.92 [2078.03, 6313.16]	2786.38 [1462.40, 4050.00]	0.085
IL-6 (median [IQR])	1.09 [0.87, 1.74]	1.53 [0.97, 1.92]	1.62 [0.81, 5.27]	0.122
β-NGF (median [IQR])	10.24 [4.39, 18.35]	14.79 [12.78, 19.79]	24.31 [12.95, 71.84]	**0.011**
IL-18 (median [IQR])	115.80 [41.46, 236.91]	105.73 [15.55, 173.78]	165.89 [93.46, 218.94]	0.403
TNFα (median [IQR])	0.00 [0.00, 38.91]	92.98 [56.81, 135.10]	127.93 [61.75, 179.53]	**<0.001**
CX3CL1 (median [IQR])	437.32 [420.50, 993.47]	517.64 [461.84, 587.34]	1361.44 [907.78, 1717.06]	**0.001**
IL-1β (median [IQR])	16.19 [6.46, 44.57]	58.89 [38.62, 104.55]	29.01 [13.92, 40.03]	**<0.001**
IFNγ (median [IQR])	9.69 [3.30, 12.57]	27.12 [17.31, 29.10]	9.35 [7.27, 23.93]	**<0.001**
CXCL8 (median [IQR])	4.49 [0.00, 10.55]	96.72 [29.88, 129.69]	27.33 [16.18, 45.05]	**<0.001**
IL-10 (median [IQR])	0.00 [0.00, 7.07]	17.05 [9.09, 28.68]	27.48 [12.85, 37.87]	**<0.001**
IL-12p70 (median [IQR])	5.44 [3.24, 6.77]	10.52 [8.78, 13.86]	15.91 [6.86, 18.10]	**<0.001**
IL-17A (median [IQR])	0.73 [0.00, 1.75]	3.16 [2.63, 4.44]	8.15 [2.58, 11.83]	**<0.001**
IL-23 (median [IQR])	4.25 [3.09, 10.03]	22.06 [12.10, 24.63]	19.88 [14.93, 27.84]	**<0.001**
IL-33 (median [IQR])	26.30 [10.21, 50.01]	83.69 [67.52, 124.72]	216.85 [107.88, 303.04]	**<0.001**

*CCL2* chemokine (C-C motif) ligand 2, *sTREM2* soluble triggering receptor expressed on myeloid cells 2, *BDNF* brain-derived neurotrophic factor, *IL-6* interleukin 6, *β-NGF* beta nerve growth factor, *IL-18* interleukin 18, *TNFα* tumor necrosis factor-alpha, *CX3CL1* chemokine (C-X3-C motif) ligand 1, *IL-1β* Interleukin-1 beta, *IFNγ* interferon gamma, *CXCL8* chemokine (C-X-C motif) ligand 8, *IL-10* interleukin 10, *IL-12p70* interleukin12p70, *IL-17A* interleukin-17A, *IL-23* interleukin 23, *IL-33* interleukin 33.

*Units of measurement:* Concentration levels of plasma cytokines, chemokines, and growth factors are expressed in picograms per milliliter (pg/mL).

*p*-values were calculated using the Kruskal–Wallis rank sum test (non-normally distributed quantitative variables).

Bold values are used to highlight statistical significance.

The cluster analysis suggests that patients with MDE have inflammatory signs, identifiable by cellular and plasma molecule characterization. Each cluster could represent a different stage of the same process or different inflammatory pathways reaching the same phenotype.

## Discussion

The present study provides a comprehensive understanding of the immune system in patients with MDE across different stages of the disease, comparing it with HC. The most novel findings include increased monocytosis with an increment of intermediate and non-classical monocyte subsets at the expense of classical monocytes, indicating a transitional activation of the monocytic population. We also observed a notable augmentation in the activation of CD4 T lymphocytes and elevated exhaustion markers in patients with active MDE compared to HC. Furthermore, there was a significant increase in the frequency of CD4^+^CD25^+^FOXP3^+^ Tregs in both active and remitted MDE patients compared to HC, which could be reflecting a compensatory anti-inflammatory immune system response. Finally, we observed increased levels of soluble markers of neuroinflammation, such as sTREM2 and IL-17A. Machine learning techniques identified a panel of biomarkers that can discriminate between patients with MDE and HC with an overall classification accuracy of 83.8%. Most of these biomarkers are related to immune cell activation. Finally, cluster analysis suggests three distinct clusters unrelated to the clinical expression of the disease.

Since the 1990s, it has been established that depression is associated with increased white blood cell count and monocytes [37], which led to the formulation of the monocytes and lymphocytes hypothesis in MDD [38]. This hypothesis suggests that alterations in these immune cells play a role in the pathophysiology of depression. A recently published meta-analysis also supported this by demonstrating an overall increase in the total number of monocytes in depressed individuals (seven studies; SMD = 0.60; 95% CI, 0.19–1.01; *p* < 0.01; *I*^2^ = 66%) [21]. Consistent with these findings, our study observed a significant monocytosis in patients experiencing an active major depressive episode (MDE). These patients exhibited a clear elevation in the median percentage and absolute number of monocytes compared to HC. These results suggest an abnormal hematopoietic response in individuals with depression, specifically an enhanced production of monocytes. We further investigated the proportion of different subtypes of circulating monocytes (classical, intermediate, and non-classical) using flow cytometry. The results indicate an expansion for the non-classical and intermediate monocytes and a reduced percentage of classical monocytes in patients with MDE compared to HC. Even though we did not find statistical differences comparing active vs. remitted MDE, more pronounced changes were observed in active conditions. These results indicate that patients with MDE are characterized by a proinflammatory status and enhanced transition to intermediate and non-classical subsets, as predicted by the monocyte transitional model of Patel et al. [39].

We have been one of the first groups to describe changes in the percentage and activation status of the three circulating monocyte subtypes in patients with severe MDD [18]. Herein, we reinforced and expanded the findings mentioned above in independent and distinct patient groups, ensuring sex and age matching and employing a simplified approach of directly measuring immune parameters in a small blood sample. This technical approach holds promise for potential translation into clinical practice, as it offers a convenient and feasible screening method.

Dysregulation of the adaptive immune system in MDE patients has been suggested, with decreased numbers of circulating T cells, an increase in the ratio of CD4^+^ relative to CD8^+^ T cells, and some immunosuppression features [16]. Our finding also suggests a dysregulation of the T-cell compartment in patients with mood disorders during MDE. While there were no significant differences in the absolute numbers of total lymphocytes, CD4 T cells, or the ratio of CD4 to CD8 T cells between MDE patients and HC, a notable increment in the activation status of CD4^+^CD69^+^, and exhausted CD4^+^PD1^+^ and CD4^+^LAG3^+^ T lymphocytes were observed in patients with active MDE compared to HC. These results indicate that CD4 lymphocyte activation is an ongoing process in patients with MDE, associated with potential exhaustion of this compartment in depression. Considering the no-identified specific antigens for mood disorders, it has recently been demonstrated the presence of naïve and memory CD4^+^ T cells which can be bystander-activated, independent of a specific antigen [40, 41]. In this sense, the increased markers of cellular activation and exhaustion in CD4 T cells among patients with MDE represent a novel concept that may explain the high comorbidity of these patients with non-psychiatric medical conditions [42], particularly autoimmune diseases [43]. Furthermore, this concept aligns with recent studies that have demonstrated a higher degree of premature T-cell aging [44].

Furthermore, there was a significant increase in the frequency of CD4^+^CD25^+^FOXP3^+^ Tregs in both active and remitted MDE patients compared to HC. The observed increase in Tregs suggests a compensatory mechanism by the immune system to counterbalance potential proinflammatory processes and regulate immune activity in MDE, as was suggested by the compensatory immune-regulatory reflex system (CIRS) concept [45]. CIRS is involved in MDD and BD by regulating the primary immune-inflammatory response, thereby contributing to spontaneous and antidepressant-promoted recovery from the acute phase of illness [45]. The simultaneously increased levels of both the pro-and anti-inflammatory cytokines are reported in the brain of MDD patients; this indicates the activity of both the IRS and CIRS in MDD. Speculation is rife that the disrupted IRS-CIRS elements might determine the onset, episodes, neuroprogressive processes, treatment response, and recovery of patients with MDD [46]. Finally, in line with the increased circulating CD4^+^CD25^+^FOXP3^+^ Tregs, elevated peripheral levels of soluble interleukin-2 receptor (sIL-2R), were also reported in MDD in an umbrella review evaluating non-genetic peripheral biomarkers for major mental disorders [15].

Patients with MDE exhibit the typical features of an ongoing inflammatory response, including increased expression of proinflammatory cytokines and their receptors, elevated acute phase reactive proteins levels, and adhesion molecules in peripheral blood, cerebrospinal fluid, and brain [14, 47]. Nonetheless, there are clear differences in the magnitude or concentration levels of these factors comparing acute inflammation in response to infections to low-grade systemic inflammation as reflected by chronic conditions. High-sensitivity CRP (hs-CRP) is a marker of acute phase response, but it has been extensively used in a specific range as a measure of low-grade inflammation in psychiatric [48] and physical conditions [49, 50]. Meta-analyses of cross-sectional studies confirm that mean concentrations of circulating hs-CRP and inflammatory cytokines such as interleukin 6 (IL-6) are higher in patients with acute depression than controls [10, 12, 51, 52]. Our findings also show increased hs-CRP levels in patients with active MDE compared to HC, but interestingly, we found that individuals with MDE in remission still exhibited elevated hs-CRP levels compared to HC, suggesting that residual inflammation may persist even after symptom improvement. Similarly, our study revealed elevated levels of IL-6 in individuals with active MDE compared to those with MDE in remission. These results suggest that IL-6 could potentially serve as a marker of ongoing inflammation during the active phase of the disease. To confirm this observation, future studies should include repeated samples from the same individual in the non-active phase. Taken together, these findings highlight the role of inflammation in MDE.

In addition to the two well-described non-specific markers of inflammation, our study found a potential novel, more specific biomarker of neuroinflammation for MDE, the soluble triggering receptor expressed on myeloid cell 2 (sTREM2). This is a protein receptor largely expressed in microglial cells in the brain. It plays a crucial role in regulating microglial function and modulating the immune response in the central nervous system (CNS). sTREM2 refers to the soluble form of this receptor, which can be measured in the cerebrospinal fluid (CSF) or peripheral blood. Changes in sTREM2 levels have been associated with the activation of microglia in neurodegenerative and neuroinflammatory diseases. A recent metanalysis of 22 observational studies, which included 5716 participants, comparing individuals with Alzheimer’s vs. controls, showed a significant increase in CSF of sTREM2 level (standardized mean difference [SMD]: 0.41, 95% confidence intervals [CI]: 0.24, 0.58, p < 0.001) [53]. This marker also increased in conditions of neuroinflammation, such as angiitis of the CNS [54] and amyotrophic lateral sclerosis (ALS) [55]. The role of sTREM2 in MDE has not been studied, and the present study is the first one reporting increased sTREM2 levels in the plasma of patients with active MDE compared with HC. Further research is needed to fully understand the role of sTREM2 in MDE and its role as a diagnostic or therapeutic target.

We also identified an increase in IL-17A (IL-17) levels, constituting an exciting soluble marker due to its association with autoimmune pathologies [56]. IL-17 is considered a signature cytokine of CD4^+^ T helper 17 (Th17) cells; however, it can also be produced by different cell types, including CD8^+^ T cells, natural Th17 cells, innate lymphoid cells (ILCs), γδ T cells, natural killer (NKT) cells, and neutrophils. Animal studies have indicated that inflammatory Th17 cells contribute to depression-like behavior [57]. Interestingly, studies have demonstrated that the administration of anti-interleukin-17A antibodies can lead to a reduction in depressive symptoms in mice [58]. In humans, there have been few studies that have examined the role of IL-17 in depression. One of the most recent studies found an increase in IL-6 and IL-17 levels in patients with a first depressive episode compared to controls [59]. In the same study, treatment with antidepressants decreased plasma levels of IL-6 and IL-17, although the latter remained elevated compared to controls [59]. Furthermore, this study also revealed that the HAMD score exhibited a moderate correlation with IL-6 and a strong correlation with IL-17 [59] which suggests that autoimmunity may play a role in the etiology or pathogenesis of depression. Our results support this idea, as we have observed significantly elevated levels of IL-17 in patients with remitted MDE and a trend (*p* = 0.06) in active MDE compared to HC. The fact that this cytokine is elevated in remitted patients may indicate that inflammation can have a chronic role in depression beyond periods of active illness, which could be highly relevant for those cases of mood disorders characterized by neuroprogression.

Upon analysis, the only significant difference in the immunological and inflammatory profile between patients with UD and BD was the higher levels of CX3CL1 in patients with UD. The CX3CL1 is implicated in inflammatory processes, neuroinflammation, immune response, oxidative stress, and neuronal function, all of which are relevant to the pathophysiology of depression. However, the findings should be carefully considered due to the study’s underpowered nature in detecting differences between these groups. Further research is warranted to elucidate the biological significance of these differences in CX3CL1 levels and their potential impact on the pathophysiology of UD and BD. The two Boruta analyses revealed different markers for discriminating between MDE patients and HC. First, comparing between MDE (active and remitted) and HC, the selected markers, including lymphocytes percentage, monocytes absolute count, classical monocytes, non-classical monocytes, intermediate monocytes, hs-CRP, CCL2, CD4^+^PD1^+^, CD4^+^LAG3^+^, and CD4^+^CD25^+^FOXP3^+^Tregs, demonstrated a significant discriminative potential. When the Random Forest model was applied to an independent test dataset, an impressive overall classification accuracy of 83.8% was achieved. Secondly, when considering the classification of active MDE, remitted MDE, and healthy controls, the Boruta algorithm identified important markers, including classical monocytes, non-classical monocytes, intermediate monocytes, monocytes absolute count, ESR, hs-CRP, CD4^+^CD69^+^, CD4^+^LAG3^+^, and CD4^+^CD25^+^FOXP3^+^ Tregs. Subsequently, the Random Forest model, trained using these selected variables, demonstrated an overall classification accuracy of 70%. Altogether, these findings highlight the potential of utilizing immune cell biomarkers to differentiate MDE patients from HC, as well as distinguish between different states of the disorder. The discriminatory power exhibited by the selected markers in the Random Forest model suggests their relevance in understanding the underlying mechanisms and aiding in the diagnostic process of MDE. Machine learning (ML) methods were employed to analyze the data, as they are known to perform well with moderate sample sizes. However, further research and validation studies with a larger sample size are warranted to explore the clinical utility and generalizability of these markers in more diverse patient populations.

The clustering analysis revealed the presence of three distinct clusters based on immunological profiles among patients with MDE. First, these results suggest that patients with MDE, regardless of whether they are experiencing or have remitted from an MDE, exhibit signs of an inflammatory state. Cluster 1 is characterized by the highest number of leukocytes, mainly given by the increment in lymphocyte count. Nonetheless, this cluster showed the lowest proinflammatory cytokines levels, probably due to a different state of the inflammation process. Cluster 3 displayed the most robust inflammatory pattern, with high levels of TNFα, CX3CL1, IL-12p70, IL-17A, IL-23, and IL-33, associated with the highest level of IL-10, as well as increased medians for β-NGF and the lowest level for BDNF. This profile is also associated with the highest absolute number and percentage of circulating monocytes as well as the lowest absolute number and percentage of circulating lymphocytes, denoting an active inflammatory process. Noteworthy, a lower percentage of individuals in Cluster 3 were receiving pharmacological treatment, indicating a potential association between the immunological profile and treatment status. Cluster 2 has some cardinal signs of more acute inflammation as the elevated levels of CCL2, which precede the monocytosis, but also increased levels of some proinflammatory cytokines such as IL-1β, IFNγ, and CXCL8. Similarly, the absolute number of monocytes is closer to an HC value, as well as the percentage of lymphocytes, suggesting as possible initiation of the inflammatory process.

Based on these results, the following questions emerge: Do the observed clusters represent distinct stages of the same underlying process, or do they indicate different inflammatory pathways that converge to produce a common phenotype? The lack of significant differences in the distribution of active or remitted MDE and the severity of depressive symptoms across the clusters suggests that they may not signify distinct stages of the same illness. However, it cannot be ruled out that these clusters represent different trajectories of the same disease, considering the limitations of our cross-sectional study design. Definitive answers to these questions will require future studies with a longitudinal design, which will provide further insights into this matter.

Peripheral inflammation in patients with MDE has been linked to an increased risk for various diseases, including cardiovascular diseases, metabolic disorders, and autoimmune conditions [60, 61]. Therefore, understanding and monitoring the inflammatory status of these patients could potentially aid in the early detection and management of not only the MDE condition but also potential associated inflammatory diseases. Furthermore, increasing evidence suggests bidirectional crosstalk between the peripheral immune system and the CNS [8]. Peripheral inflammation can influence neuroinflammatory processes and neurotransmitter systems within the brain, which may contribute to the pathophysiology of psychiatric disorders such as depression. Identifying specific markers of peripheral inflammation associated with MDE may provide insights into the underlying mechanisms linking peripheral and central inflammation. This, in turn, could potentially lead to the development of novel treatment strategies targeting immune dysregulation in psychiatric disorders for more effective interventions.

Our study demonstrates several noteworthy strengths. Firstly, we assessed changes in cellular levels of the monocyte compartment and T cells, considering a specific plasma cytokine milieu. This allowed us to establish a distinct profile for MDE patients, defining subtypes of the condition. This approach fills a critical gap in the literature, as this area has received inadequate attention thus far. Secondly, our standardized methodology employed three cocktails of antibodies with a minimal blood sample volume of only 100 µL each. This approach allowed us to accurately measure the proportion and activation of monocytes, the proportion of CD4 to CD8 lymphocytes, and Tregs, as well as the activation and exhaustion of T cells, utilizing direct blood staining. Such an approach holds promise for rapid translation into clinical practice. Thirdly, our study is a multicenter investigation that carefully matched participants based on age and sex, two variables known to significantly influence the immune system. By controlling for these factors, we strengthened the validity and generalizability of our results. Finally, our rigorous patient selection process excluded individuals with known causes of inflammation or immune system activation, ensuring the focus remained on the specific MDE condition. Additionally, we included patients at various stages of the disease, a novel aspect not previously explored in MDE immunotyping studies.

Some limitations of the study need to be acknowledged. Firstly, we measured the immune cell profile in peripheral blood, which may not fully reflect the immune activity in the central nervous system (CNS). However, evidence suggests that inflammatory factors originating in the blood can reach the CNS through various pathways, including passive or active transport across the blood-brain barrier, immune cell transmigration, and vagal nerve signaling [8]. While peripheral blood analysis provides valuable insights, it is important to recognize the potential disparities between peripheral and CNS immune responses. Another significant limitation is that most participants received psychopharmacological treatment at the time of inclusion. It is well-known that many psychotropic medications can impact the immune system, potentially confounding the interpretation of immunological findings. Furthermore, our study focused exclusively on individuals with MDE, which could be a limitation as MDD and BP may exhibit distinct immune profiles. However, in clinical practice, MDE is the most commonly encountered presentation, and there are currently no precise indicators that reliably classify between these two groups. Therefore, we included MDD and BP patients to explore whether immunological markers could provide insights into their shared pathophysiology. Another limitation of our study is that the results should be considered exploratory. The p-values reported for the comparison analyses of clinical and biological variables were adjusted for multiple comparisons among groups only for those variables with a statistically significant difference in the bivariate analysis. Finally, case–control studies do not allow to infer causal relationships due to the inherent design limitations. However, such studies play a crucial role in establishing a foundation for future longitudinal investigations.

Despite these limitations, our study provides valuable insights into the immunological aspects of MDE providing a global view of the phenomenon, and analyzing both the humoral and the innate and adaptive cellular components. Further research is needed to fully understand the implications of these immune alterations in a longitudinal process to pave the way for potential advancements in clinical practice.

### Supplementary information

Supplemental Material

## Data Availability

The data supporting this study’s findings are available from the corresponding authors.
