# Supplementary material for: Decoding the inflammatory signature of the major depressive episode: insights from peripheral immunophenotyping in active and remitted condition, a case–control study
Source: Transl Psychiatry. 2024 Jun 12;14:254. doi: 10.1038/s41398-024-02902-2 (PMC11169351; doi:10.1038/s41398-024-02902-2)
Supplement: Supplementary file 1 — Supplemental Material [file 41398_2024_2902_MOESM1_ESM.docx]

**Supplementary Table S1**: Baseline clinical characteristics of the patients with MDE included in the study

| **Variable** | **Active MDE** | **Remitted MDE** | **p-value** |
| --- | --- | --- | --- |
| Age of onset form first symptoms (median [IQR]) | 20.00 [16.00, 31.50] | 21.00 [17.00, 30.00] | 0.669 |
| Age of onset from first treatment (mean (SD)) | 29.95 (13.47) | 30.51 (11.82) | 0.845 |
| Age of onset from first hospitalization (mean (SD)) | 34.73 (17.83) | 31.28 (8.27) | 0.483 |
| Duration of illness from first symptoms (median [IQR]) | 17.00 [10.00, 24.00] | 13.00 [8.00, 22.00] | 0.343 |
| Duration of illness from first treatment (median [IQR]) | 12.50 [3.25, 18.00] | 10.00 [3.00, 14.00] | 0.584 |
| Duration of illness from first hospitalization (median [IQR]) | 3.00 [1.00, 13.00] | 13.00 [4.25, 14.00] | 0.154 |
| Hospitalizations for mania (median [IQR]) | 0.00 [0.00, 0.00] | 0.00 [0.00, 2.00] | **0.001** |
| Hospitalization for depression (median [IQR]) | 0.00 [0.00, 1.00] | 0.00 [0.00, 0.00] | 0.609 |
| Total hospitalizations (median [IQR]) | 0.00 [0.00, 1.00] | 0.00 [0.00, 2.25] | 0.057 |
| Total weeks hospitalized (median [IQR]) | 0.00 [0.00, 1.00] | 0.00 [0.00, 4.00] | 0.190 |
| Total weeks hospitalized per year ill (median [IQR]) | 0.00 [0.00, 0.00] | 0.00 [0.00, 0.00] | 0.788 |
| Episodes of mania (median [IQR]) | 0.00 [0.00, 1.00] | 1.00 [0.00, 3.25] | **0.040** |
| Episodes of depression (median [IQR]) | 3.00 [2.00, 6.00] | 2.00 [1.00, 4.25] | 0.060 |
| Total number of episodes (median [IQR]) | 4.00 [2.00, 8.00] | 4.00 [2.75, 6.00] | 0.786 |

**Supplementary Table S2.** Comparison of psychotropic medication among patients with MDE

| **Psychotropic medication** | **Active MDE** | **Remitted MDE** | **p-value** |
| --- | --- | --- | --- |
| Antidepressant (Yes, %) | 23 (59.0) | 14 (35.0) | 0.056 |
| Mood stabilizers (Yes, %) | 13 (33.3) | 24 (60.0) | **0.032** |
| Antipsychotics (Yes, %) | 18 (46.2) | 21 (52.5) | 0.735 |
| Benzodiazepines and hypnotics (Yes, %) | 25 (64.1) | 18 (45.0) | 0.139 |

**Supplementary Table S3**. Analysis and comparation of T Cell compartment by flow cytometry

| **Variable** | **Active disease** | **Inactive disease** | **Control** | **p-value** |
| --- | --- | --- | --- | --- |
| Tcell CD3+CD4+ (median [IQR]) | 58.50 [50.30, 64.50] | 57.90 [49.30, 63.25] | 60.25 [54.05, 65.83] | 0.614 |
| Tcell CD3+CD8+ (median [IQR]) | 27.30 [23.10, 33.85] | 29.10 [24.75, 32.65] | 28.55 [26.02, 32.17] | 0.725 |
| Ratio CD4/CD8 (median [IQR]) | 1.92 [1.67, 2.63] | 2.00 [1.49, 2.40] | 2.05 [1.78, 2.43] | 0.672 |
| CD4+CD44+ (median [IQR]) | 48.05 [36.02, 62.33] | 51.10 [29.55, 63.95] | 42.00 [32.25, 60.78] | 0.868 |

**Supplementary Table S4.** Pairs of Variables with Correlations Greater than 0.50 (in absolute value)

| **Variable 1** | **Variable 2** | **Correlation** |
| --- | --- | --- |
| IL-10 | IL-12p70 | 0.78 |
| IL-10 | IL-33 | 0.73 |
| IL-1β | IFNγ | 0.70 |
| IL-17A | IL-33 | 0.67 |
| IL-23 | IL-33 | 0.67 |
| IL-12p70 | IL-33 | 0.63 |
| IL-12p70 | IL-23 | 0.62 |
| IL-17A | IL-23 | 0.59 |
| IL-12p70 | IL-17A | 0.59 |
| IL-10 | IL-23 | 0.59 |
| TNFα | IL-23 | 0.58 |
| TNFα | IL-10 | 0.57 |
| IFNγ | IL-17A | 0.57 |
| IL-10 | IL-17A | 0.55 |
| CXCL8 | IL-23 | 0.53 |
| CXCL8 | IL-12p70 | 0.53 |
| IFNγ | IL-23 | 0.52 |
| CCL2 | BDNF | 0.51 |
| TNFα | IL-12p70 | 0.51 |
| IL-1β | IL-12p70 | 0.51 |

**Supplementary Table S5**. Comparison of Sociodemographic characteristics among clusters

| **Variable** | **1** | **2** | **3** | **p-value** |
| --- | --- | --- | --- | --- |
| n | 44 | 20 | 15 |  |
| Group (%) |  |  |  | 0.623 |
| Active disease | 23 (52.3) | 8 (40.0) | 8 (53.3) |  |
| Inactive disease | 21 (47.7) | 12 (60.0) | 7 (46.7) |  |
| Age (median [IQR]) | 43.00 [35.00, 52.25] | 34.00 [27.75, 53.00] | 40.00 [36.50, 47.00] | 0.544 |
| Gender = Male (%) | 16 (36.4) | 5 (25.0) | 3 (20.0) | 0.410 |
| Civil status (%) |  |  |  | 0.472 |
| Married/Living with a partner | 12 (27.3) | 2 (10.0) | 2 (13.3) |  |
| Separated/Divorced/Widower | 10 (22.7) | 6 (30.0) | 3 (20.0) |  |
| Single | 22 (50.0) | 12 (60.0) | 10 (66.7) |  |
| Scholarship (%) |  |  |  | 0.295 |
| None | 0 ( 0.0) | 0 ( 0.0) | 0 ( 0.0) |  |
| Incomplete primary | 0 ( 0.0) | 0 ( 0.0) | 0 ( 0.0) |  |
| Complete primary | 5 (11.4) | 0 ( 0.0) | 0 ( 0.0) |  |
| Incomplete high school | 9 (20.5) | 4 (20.0) | 2 (13.3) |  |
| Complete high school | 6 (13.6) | 2 (10.0) | 3 (20.0) |  |
| Incomplete college | 17 (38.6) | 8 (40.0) | 3 (20.0) |  |
| Complete college | 7 (15.9) | 6 (30.0) | 7 (46.7) |  |
| Employment status = Unemployed/Retired (%) | 22 (50.0) | 8 (40.0) | 6 (40.0) | 0.675 |
| Diagnostic summary (%) |  |  |  | 0.112 |
| Bipolar disorder I | 15 (34.1) | 10 (50.0) | 2 (13.3) |  |
| Bipolar disorder II | 7 (15.9) | 1 ( 5.0) | 4 (26.7) |  |
| Major depressive disorder | 22 (50.0) | 8 (40.0) | 9 (60.0) |  |
| Non-specific Bipolar disorder | 0 ( 0.0) | 1 ( 5.0) | 0 ( 0.0) |  |
| None depressive disorder | 0 ( 0.0) | 0 ( 0.0) | 0 ( 0.0) |  |
| HAM-D Total score (median [IQR]) | 9.50 [4.00, 15.50] | 6.00 [1.00, 10.50] | 8.00 [5.50, 14.50] | 0.398 |
| On pshycopharmacological treatment = Yes (%) | 41 (95.3) | 19 (95.0) | 9 (60.0) | **0.001** |
| Total number of ACEs (median [IQR]) | 4.50 [2.00, 6.00] | 2.50 [2.00, 5.00] | 3.00 [2.50, 5.50] | 0.482 |
| IPAQ (%) |  |  |  | **0.039** |
| Low | 23 (52.3) | 9 (45.0) | 9 (60.0) |  |
| Median | 8 (18.2) | 3 (15.0) | 6 (40.0) |  |
| High | 13 (29.5) | 8 (40.0) | 0 ( 0.0) |  |
| Suicide ideation month = Yes (%) | 14 (45.2) | 5 (41.7) | 2 (14.3) | 0.130 |
| Suicide Behaviour month = Yes (%) | 1 ( 2.3) | 1 ( 5.0) | 0 ( 0.0) | 0.693 |
| Suicide ideation life = Yes (%) | 33 (94.3) | 15 (93.8) | 12 (85.7) | 0.701 |
| Suicide Behaviour life = Yes (%) | 15 (34.1) | 10 (50.0) | 4 (26.7) | 0.316 |
| Number of Stressful Life Events (mean (SD)) | 10.52 (5.51) | 9.80 (3.25) | 10.73 (4.77) | 0.821 |
| Stressful Life Events Total Score (mean (SD)) | 327.05 (176.47) | 303.90 (122.85) | 330.00 (113.37) | 0.834 |
| Age of onset form first symptoms (median [IQR]) | 24.50 [17.00, 33.50] | 19.00 [14.75, 29.75] | 20.00 [19.00, 25.00] | 0.291 |
| Age of onset from first treatment (mean (SD)) | 32.30 (12.07) | 28.70 (15.85) | 26.07 (7.15) | 0.226 |
| Age of onset from first hospitalization (median [IQR]) | 24.00 [0.00, 33.00] | 18.00 [0.00, 30.00] | 0.00 [0.00, 0.75] | 0.116 |
| Duration of illness from first symptoms (median [IQR]) | 14.00 [9.50, 21.00] | 15.00 [6.00, 20.00] | 19.00 [11.50, 25.50] | 0.668 |
| Duration of illness from first treatment (median [IQR]) | 10.00 [3.00, 14.75] | 9.00 [2.50, 15.00] | 13.00 [4.75, 22.75] | 0.386 |
| Duration of illness from first hospitalization (median [IQR]) | 0.00 [0.00, 10.00] | 0.00 [0.00, 8.00] | 0.00 [0.00, 0.00] | 0.500 |
| Hospitalizations for mania (median [IQR]) | 0.00 [0.00, 0.75] | 0.00 [0.00, 0.50] | 0.00 [0.00, 0.00] | 0.227 |
| Hospitalization for depression (median [IQR]) | 0.00 [0.00, 0.00] | 0.00 [0.00, 0.50] | 0.00 [0.00, 0.00] | 0.914 |
| Total hospitalizations (median [IQR]) | 0.00 [0.00, 1.50] | 0.00 [0.00, 1.00] | 0.00 [0.00, 0.00] | 0.243 |
| Total weeks hospitalized (median [IQR]) | 0.00 [0.00, 8.00] | 0.00 [0.00, 2.00] | 0.00 [0.00, 0.00] | 0.469 |
| Total weeks hospitalized per year ill (median [IQR]) | 0.00 [0.00, 0.00] | 0.00 [0.00, 0.00] | 0.00 [0.00, 0.00] | 0.712 |
| Episodes of mania (median [IQR]) | 0.00 [0.00, 3.25] | 1.00 [0.00, 3.25] | 0.00 [0.00, 0.00] | 0.070 |
| Episodes of depression (median [IQR]) | 3.00 [1.00, 5.25] | 2.50 [1.75, 5.00] | 3.00 [2.00, 4.00] | 0.658 |
| Total number of episodes (median [IQR]) | 5.00 [3.00, 8.25] | 4.00 [2.00, 7.00] | 3.00 [2.00, 4.50] | 0.115 |
| Weight (median [IQR]) | 71.00 [61.75, 85.00] | 63.00 [58.83, 75.00] | 68.50 [60.50, 75.55] | 0.391 |
| Height (mean (SD)) | 166.02 (9.11) | 163.50 (6.44) | 164.80 (10.21) | 0.559 |
| BMI (median [IQR]) | 25.75 [22.87, 30.82] | 24.60 [21.95, 30.65] | 25.40 [22.55, 26.35] | 0.722 |
| Amount of cigarettes per day (median [IQR]) | 0.50 [0.00, 15.75] | 0.00 [0.00, 0.50] | 0.00 [0.00, 3.00] | 0.184 |
| Alcohol use = Yes (%) | 13 (29.5) | 7 (35.0) | 8 (53.3) | 0.250 |

**Supplementary Table S6**. Comparison of monocytes subtypes and T cells compartment among clusters

| **Variable** | **1** | **2** | **3** | **p-value** |
| --- | --- | --- | --- | --- |
| Classic monocytes (median [IQR]) | 74.40 [66.30, 83.95] | 68.80 [62.82, 80.60] | 74.20 [64.30, 77.30] | 0.275 |
| Intermediate monocytes (median [IQR]) | 11.15 [7.44, 17.68] | 13.45 [9.55, 18.62] | 12.10 [9.40, 16.20] | 0.670 |
| Non classic monocytes (median [IQR]) | 7.73 [5.39, 12.30] | 9.77 [5.26, 18.85] | 8.70 [5.72, 11.24] | 0.617 |
| Tcell CD3+CD4+ (median [IQR]) | 59.25 [55.08, 64.95] | 61.10 [56.48, 65.82] | 46.60 [42.35, 49.75] | **<0.001** |
| Tcell CD3+CD8+ (median [IQR]) | 28.25 [24.05, 35.50] | 28.50 [26.30, 32.08] | 28.30 [24.85, 31.90] | 0.988 |
| CD4+CD44+ (mean (SD)) | 49.03 (18.69) | 39.67 (22.32) | 57.88 (19.55) | 0.053 |
| CD4+CD69+ (median [IQR]) | 4.56 [2.15, 8.80] | 2.19 [1.41, 3.50] | 2.80 [1.48, 8.68] | 0.095 |
| CD4+PD1+ (median [IQR]) | 8.12 [4.60, 20.85] | 5.84 [2.14, 9.39] | 8.45 [5.56, 8.98] | 0.237 |
| CD4+LAG3+ (median [IQR]) | 5.00 [1.67, 13.03] | 1.31 [0.56, 5.30] | 4.15 [1.63, 5.53] | **0.025** |
| Tregs CD4+CD25+ FOXP3+ (median [IQR]) | 5.78 [2.74, 9.97] | 5.80 [3.62, 7.93] | 4.04 [2.90, 7.12] | 0.629 |

**Supplementary Table S7.** Comparison of psychotropic medication among clusters

| **Variable** | **1** | **2** | **3** | **p-value** |
| --- | --- | --- | --- | --- |
| Antidepressant = Yes (%) | 22 (50.0) | 8 (40.0) | 7 (46.7) | 0.250 |
| Mood stabilizers = Yes (%) | 21 (47.7) | 13 (65.0) | 3 (20.0) | **0.027** |
| Antipsychotics = Yes (%) | 26 (59.1) | 11 (55.0) | 2 (13.3) | **0.007** |
| Benzodiazepines and hypnotics = Yes (%) | 25 (56.8) | 14 (70.0) | 4 (26.7) | **0.035** |

**Supplementary Table S8**: Baseline clinical characteristics of cases included in the study, by diagnosis.

| **Variable** | **BD** | **UD** | **p-value** |
| --- | --- | --- | --- |
| n | 40 | 81 |  |
| Age (median [IQR]) | 40.50 [34.00, 51.25] | 42.00 [30.00, 52.00] | 0.873 |
| Gender = Male (%) | 13 (32.5) | 25 (30.9) | 1.000 |
| Civil status (%) |  |  | 0.394 |
| Married/Living with a partner | 7 (17.5) | 22 (27.2) |  |
| Separated/Divorced/Widower | 10 (25.0) | 14 (17.3) |  |
| Single | 23 (57.5) | 45 (55.6) |  |
| Scholarship (%) |  |  | 0.169 |
| None | 0 ( 0.0) | 0 ( 0.0) |  |
| Incomplete primary | 0 ( 0.0) | 0 ( 0.0) |  |
| Complete primary | 1 ( 2.5) | 4 ( 4.9) |  |
| Incomplete high school | 8 (20.0) | 8 ( 9.9) |  |
| Complete high school | 7 (17.5) | 6 ( 7.4) |  |
| Incomplete college | 11 (27.5) | 33 (40.7) |  |
| Complete college | 13 (32.5) | 30 (37.0) |  |
| Employment status = Unemployed/Retired (%) | 20 (50.0) | 21 (25.9) | **0.015** |
| HAM-D Total score (median [IQR]) | 5.50 [1.00, 11.50] | 2.00 [0.00, 9.00] | 0.064 |
| On pshycopharmacological treatment = Yes (%) | 38 (97.4) | 31 (39.2) | **<0.001** |
| Total number of ACEs (median [IQR]) | 3.50 [2.00, 6.00] | 2.00 [0.00, 4.00] | **0.001** |
| IPAQ (%) |  |  | 0.960 |
| Low | 20 (50.0) | 40 (49.4) |  |
| Median | 9 (22.5) | 20 (24.7) |  |
| High | 11 (27.5) | 21 (25.9) |  |
| Suicide ideation month = Yes (%) | 9 (39.1) | 12 (31.6) | 0.587 |
| Suicide Behaviour month = Yes (%) | 1 ( 2.5) | 1 ( 1.2) | 1.000 |
| Suicide ideation life = Yes (%) | 29 (93.5) | 31 (81.6) | 0.171 |
| Suicide Behaviour life = Yes (%) | 19 (47.5) | 10 (12.3) | **<0.001** |
| Number of Stressful Life Events (median [IQR]) | 10.00 [6.75, 15.00] | 9.00 [6.00, 12.00] | 0.341 |
| Stressful Life Events Total Score (median [IQR]) | 276.50 [181.50, 469.00] | 246.00 [165.00, 386.00] | 0.129 |
| Weight (median [IQR]) | 70.00 [60.00, 88.75] | 67.00 [60.00, 77.50] | 0.314 |
| Height (mean (SD)) | 166.55 (9.02) | 165.81 (8.68) | 0.666 |
| BMI (median [IQR]) | 25.75 [21.75, 31.20] | 24.50 [22.60, 27.70] | 0.323 |
| Amount of cigarettes per day (median [IQR]) | 0.00 [0.00, 16.25] | 0.00 [0.00, 1.00] | **0.016** |
| Alcohol use = Yes (%) | 12 (30.0) | 47 (58.8) | **0.006** |

BD: Bipolar Depression, UP: Unipolar Depression

**Supplementary Table S9 :** Baseline disease-related characteristics of cases included in the study, by diagnosis.

| **Variable** | **BD** | **UD** | **p-value** |
| --- | --- | --- | --- |
| Age of onset form first symptoms (median [IQR]) | 21.50 [17.00, 29.25] | 20.00 [16.50, 38.50] | 0.300 |
| Age of onset from first treatment (mean (SD)) | 27.02 (10.90) | 33.70 (13.47) | **0.019** |
| Age of onset from first hospitalization (mean (SD)) | 30.20 (11.10) | 47.50 (12.01) | **0.008** |
| Duration of illness from first symptoms (median [IQR]) | 17.00 [13.00, 26.00] | 11.50 [4.00, 19.50] | **0.010** |
| Duration of illness from first treatment (median [IQR]) | 13.00 [9.00, 19.00] | 8.00 [1.50, 12.50] | **0.001** |
| Duration of illness from first hospitalization (median [IQR]) | 13.00 [3.00, 14.00] | 0.50 [0.00, 4.00] | **0.045** |
| Hospitalizations for mania (median [IQR]) | 0.00 [0.00, 2.00] | 0.00 [0.00, 0.00] | **<0.001** |
| Hospitalization for depression (median [IQR]) | 0.00 [0.00, 1.00] | 0.00 [0.00, 0.00] | **<0.001** |
| Total hospitalizations (median [IQR]) | 1.00 [0.00, 3.00] | 0.00 [0.00, 0.00] | **<0.001** |
| Total weeks hospitalized (median [IQR]) | 2.50 [0.00, 16.00] | 0.00 [0.00, 0.00] | **<0.001** |
| Total weeks hospitalized per year ill (median [IQR]) | 0.00 [0.00, 0.85] | 0.00 [0.00, 0.00] | **<0.001** |
| Episodes of mania (median [IQR]) | 3.00 [1.00, 5.00] | 0.00 [0.00, 0.00] | **<0.001** |
| Episodes of depression (median [IQR]) | 3.50 [1.75, 6.00] | 2.00 [0.00, 3.00] | **0.001** |
| Total number of episodes (median [IQR]) | 6.50 [4.00, 10.25] | 2.00 [0.00, 3.00] | **<0.001** |

BD: Bipolar Depression, UD: Unipolar Depression

**Supplementary Table S10 :** Biochemical parameters of study participants, by diagnosis.

| **Variable** | **BD** | **UD** | **p-value** |
| --- | --- | --- | --- |
| Hematocrit (median [IQR]) | 42.40 [39.55, 45.05] | 41.85 [39.60, 43.55] | 0.247 |
| Hemoglobin (median [IQR]) | 13.90 [12.95, 14.85] | 13.80 [12.90, 14.50] | 0.423 |
| Erythrocytes (mean (SD)) | 4.76 (0.54) | 4.77 (0.46) | 0.953 |
| MCV (median [IQR]) | 89.93 [87.03, 93.35] | 86.72 [84.07, 89.72] | **0.001** |
| MCH (median [IQR]) | 29.63 [28.61, 30.08] | 28.80 [27.79, 29.93] | **0.026** |
| MCHC (median [IQR]) | 32.96 [32.50, 33.77] | 32.99 [32.50, 33.80] | 0.754 |
| Leukocytes (median [IQR]) | 7.10 [5.60, 8.90] | 6.65 [5.59, 8.07] | 0.420 |
| Segmented neutrophils Percentage (median [IQR]) | 60.20 [51.00, 66.00] | 56.00 [51.00, 62.00] | 0.146 |
| Segmented neutrophils absolute count (median [IQR]) | 3.84 [3.01, 5.46] | 3.79 [2.94, 4.76] | 0.385 |
| Lymphocytes Percentage (mean (SD)) | 33.44 (10.01) | 36.49 (8.37) | 0.111 |
| Lymphocytes absolute count (median [IQR]) | 2.13 [1.87, 2.47] | 2.44 [2.06, 2.77] | 0.131 |
| Monocytes Percentage (median [IQR]) | 5.00 [3.00, 8.00] | 4.00 [2.00, 7.00] | 0.108 |
| Monocytes absolute count (median [IQR]) | 0.38 [0.21, 0.53] | 0.25 [0.17, 0.44] | 0.053 |
| Eosinophils Percentage (median [IQR]) | 2.00 [2.00, 2.60] | 2.00 [1.00, 2.30] | 0.544 |
| Eosinophils absolute count (median [IQR]) | 0.14 [0.10, 0.22] | 0.13 [0.09, 0.20] | 0.444 |
| Basophils Percentage (median [IQR]) | 0.00 [0.00, 0.00] | 0.00 [0.00, 0.00] | 0.824 |
| Basophils absolute count (median [IQR]) | 0.00 [0.00, 0.00] | 0.00 [0.00, 0.00] | 0.770 |
| ESR (median [IQR]) | 10.00 [7.00, 18.00] | 10.00 [7.00, 14.00] | 0.477 |
| Urea (median [IQR]) | 29.50 [26.00, 37.00] | 29.00 [23.00, 35.00] | 0.388 |
| Creatinine (median [IQR]) | 8.10 [0.98, 10.35] | 7.90 [1.02, 9.57] | 0.637 |
| GOT (median [IQR]) | 23.50 [18.00, 30.75] | 23.50 [17.25, 30.00] | 0.892 |
| GPT (median [IQR]) | 26.50 [16.00, 36.00] | 24.00 [16.25, 31.00] | 0.623 |
| Alkaline phosphatase (median [IQR]) | 148.50 [87.00, 205.00] | 155.00 [108.50, 185.00] | 0.796 |
| Total Bilirubin (median [IQR]) | 0.48 [0.34, 0.69] | 0.55 [0.42, 0.77] | 0.216 |
| Sodium (mean (SD)) | 138.26 (3.39) | 138.05 (3.45) | 0.752 |
| Potassium (median [IQR]) | 4.30 [4.00, 4.70] | 4.30 [4.00, 4.55] | 0.357 |
| Chloride (median [IQR]) | 99.00 [91.50, 101.20] | 94.00 [89.00, 100.00] | **0.015** |
| HS CRP (median [IQR]) | 1.86 [0.56, 4.97] | 0.95 [0.39, 3.57] | 0.124 |

BD: Bipolar Depression, UD: Unipolar Depression

**Supplementary Table S11:** Percentage of classical, intermediate, and non-classical monocytes in study participants, by diagnosis.

| **Variable** | **BD** | **UD** | **p-value** |
| --- | --- | --- | --- |
| Classic monocytes (median [IQR]) | 78.05 [64.78, 84.40] | 81.10 [72.40, 86.50] | 0.104 |
| Intermediate monocytes (median [IQR]) | 10.90 [7.04, 17.68] | 7.77 [5.76, 13.60] | 0.124 |
| Non classic monocytes (median [IQR]) | 6.83 [4.60, 13.52] | 5.91 [3.43, 9.29] | 0.080 |

BD: Bipolar Depression, UD: Unipolar Depression

**Supplementary Table S12:** Plasma level of cytokines, chemokines and neurotrophic factors of study participants, by diagnosis**.**

| **Variable** | **BD** | **UD** | **p-value** |
| --- | --- | --- | --- |
| CCL2 (median [IQR]) | 53.22 [35.44, 91.60] | 51.23 [40.90, 66.62] | 0.613 |
| sTREM2 (median [IQR]) | 1009.40 [737.48, 1270.68] | 886.54 [672.83, 1192.81] | 0.380 |
| BDNF (median [IQR]) | 3914.20 [2562.95, 5958.47] | 4794.16 [2668.18, 7037.34] | 0.307 |
| IL-6 (median [IQR]) | 1.33 [0.90, 2.11] | 1.29 [0.87, 1.94] | 0.938 |
| β-NGF (median [IQR]) | 10.51 [6.75, 19.40] | 13.58 [5.03, 21.24] | 0.976 |
| IL-18 (median [IQR]) | 178.89 [41.27, 248.18] | 108.14 [48.28, 160.84] | 0.109 |
| TNFα (median [IQR]) | 57.13 [0.00, 94.70] | 31.12 [0.00, 108.92] | 0.735 |
| CX3CL1 (median [IQR]) | 460.47 [422.65, 943.25] | 783.34 [436.15, 1356.86] | **0.022** |
| IL-1β (median [IQR]) | 31.65 [11.52, 76.84] | 21.45 [11.87, 42.90] | 0.144 |
| IFNγ (median [IQR]) | 13.51 [4.95, 27.12] | 10.89 [6.65, 17.20] | 0.265 |
| CXCL8 (median [IQR]) | 10.52 [0.88, 37.36] | 12.66 [5.65, 40.87] | 0.567 |
| IL-10 (median [IQR]) | 5.65 [0.00, 18.50] | 6.23 [0.00, 14.96] | 0.964 |
| IL-12p70 (median [IQR]) | 6.97 [3.70, 11.12] | 6.66 [5.01, 11.12] | 0.871 |
| IL-17A (median [IQR]) | 1.91 [0.69, 2.99] | 1.40 [0.02, 3.45] | 0.405 |
| IL-23 (median [IQR]) | 10.34 [3.43, 21.06] | 9.52 [4.15, 15.84] | 0.859 |
| IL-33 (median [IQR]) | 63.76 [22.28, 100.01] | 50.54 [16.69, 97.92] | 0.523 |

BD: Bipolar Depression, UD: Unipolar Depression

**Supplementary Table S13** : T-cell values of study participants, by diagnosis.

| **Variable** | **BD** | **UD** | **p-value** |
| --- | --- | --- | --- |
| Tcell CD3+CD4+ (median [IQR]) | 59.55 [48.83, 65.70] | 58.50 [52.32, 62.57] | 0.826 |
| Tcell CD3+CD8+ (median [IQR]) | 28.95 [24.25, 34.95] | 28.25 [24.40, 32.75] | 0.717 |
| Ratio CD4/CD8 (median [IQR]) | 2.01 [1.39, 2.44] | 2.01 [1.69, 2.49] | 0.508 |
| CD4+CD44+ (median [IQR]) | 45.45 [30.33, 60.77] | 47.30 [33.25, 63.35] | 0.993 |
| CD4+CD69+ (median [IQR]) | 2.44 [1.73, 4.72] | 2.66 [1.08, 5.50] | 0.883 |
| CD4+PD1+ (median [IQR]) | 6.30 [2.14, 11.82] | 5.94 [3.85, 8.92] | 0.592 |
| CD4+LAG3+ (median [IQR]) | 2.01 [0.88, 7.29] | 2.27 [1.08, 5.20] | 0.966 |
| Tregs CD4+CD25+ FOXP3+ (median [IQR]) | 5.39 [2.50, 9.35] | 3.31 [2.04, 6.84] | 0.283 |

BD: Bipolar Depression, UD: Unipolar Depression

**Supplementary Table S14:** Baseline clinical characteristics of cases included in the study, by sex.

| **Variable** | **Female** | **Male** | **p-value** |
| --- | --- | --- | --- |
| n | 83 | 38 |  |
| Age (median [IQR]) | 43.00 [31.50, 52.00] | 37.00 [28.50, 49.25] | 0.134 |
| Civil status (%) |  |  | 0.725 |
| Married/Living with a partner | 20 (24.1) | 9 ( 23.7) |  |
| Separated/Divorced/Widower | 18 (21.7) | 6 ( 15.8) |  |
| Single | 45 (54.2) | 23 ( 60.5) |  |
| Scholarship (%) |  |  | 0.138 |
| Complete primary | 5 ( 6.0) | 0 ( 0.0) |  |
| Incomplete high school | 10 (12.0) | 6 ( 15.8) |  |
| Complete high school | 8 ( 9.6) | 5 ( 13.2) |  |
| Incomplete college | 26 (31.3) | 18 ( 47.4) |  |
| Complete college | 34 (41.0) | 9 ( 23.7) |  |
| Employment status = Unemployed/Retired (%) | 27 (32.5) | 14 ( 36.8) | 0.796 |
| Diagnostic summary (%) |  |  | 0.712 |
| Bipolar disorder I | 19 (22.9) | 8 ( 21.1) |  |
| Bipolar disorder II | 8 ( 9.6) | 4 ( 10.5) |  |
| Major depressive disorder | 28 (33.7) | 11 ( 28.9) |  |
| Non-specific Bipolar disorder | 0 ( 0.0) | 1 ( 2.6) |  |
| None depressive disorder | 28 (33.7) | 14 ( 36.8) |  |
| HAM-D Total score (median [IQR]) | 3.00 [0.00, 11.00] | 3.50 [0.00, 8.25] | 0.589 |
| On pshycopharmacological treatment = Yes (%) | 46 (57.5) | 23 ( 60.5) | 0.911 |
| Total number of ACEs (median [IQR]) | 3.00 [1.00, 6.00] | 1.00 [0.00, 3.00] | **0.005** |
| IPAQ (%) |  |  | 0.065 |
| Low | 47 (56.6) | 13 ( 34.2) |  |
| Median | 18 (21.7) | 11 ( 28.9) |  |
| High | 18 (21.7) | 14 ( 36.8) |  |
| Suicide ideation month = Yes (%) | 14 (31.1) | 7 ( 43.8) | 0.376 |
| Suicide Behaviour month = Yes (%) | 1 ( 1.2) | 1 ( 2.6) | 0.531 |
| Suicide ideation life = Yes (%) | 43 (84.3) | 17 ( 94.4) | 0.428 |
| Suicide Behaviour life = Yes (%) | 17 (20.5) | 12 ( 31.6) | 0.272 |
| Number of Stressful Life Events (median [IQR]) | 9.00 [6.00, 13.00] | 10.00 [7.00, 12.75] | 0.440 |
| Stressful Life Events Total Score (median [IQR]) | 250.00 [158.00, 398.00] | 296.50 [180.25, 385.25] | 0.647 |
| Weight (median [IQR]) | 64.50 [58.00, 73.50] | 80.50 [72.00, 94.00] | **<0.001** |
| Height (mean (SD)) | 162.42 (6.91) | 174.00 (6.98) | **<0.001** |
| BMI (median [IQR]) | 24.20 [21.90, 28.40] | 26.25 [23.27, 30.30] | 0.061 |
| Amount of cigarettes per day (median [IQR]) | 0.00 [0.00, 3.50] | 0.00 [0.00, 5.25] | 0.911 |
| Alcohol use = Yes (%) | 39 (47.6) | 20 ( 52.6) | 0.749 |

**Supplementary Table S15 :** Baseline disease-related characteristics of cases included in the study, by sex.

| **Variable** | **Female** | **Male** | **p-value** |
| --- | --- | --- | --- |
| Age of onset form first symptoms (median [IQR]) | 20.00 [16.50, 31.50] | 22.50 [17.00, 30.00] | 0.583 |
| Age of onset from first treatment (mean (SD)) | 29.92 (12.52) | 30.92 (12.95) | 0.751 |
| Age of onset from first hospitalization (mean (SD)) | 32.84 (13.09) | 32.10 (12.19) | 0.883 |
| Duration of illness from first symptoms (median [IQR]) | 16.00 [10.00, 25.75] | 14.00 [8.50, 17.50] | 0.191 |
| Duration of illness from first treatment (median [IQR]) | 11.50 [3.25, 16.00] | 9.00 [2.50, 14.50] | 0.433 |
| Duration of illness from first hospitalization (median [IQR]) | 11.00 [2.50, 13.50] | 8.00 [1.50, 14.00] | 0.872 |
| Hospitalizations for mania (median [IQR]) | 0.00 [0.00, 0.00] | 0.00 [0.00, 0.00] | 0.155 |
| Hospitalization for depression (median [IQR]) | 0.00 [0.00, 0.00] | 0.00 [0.00, 1.00] | 0.087 |
| Total hospitalizations (median [IQR]) | 0.00 [0.00, 1.00] | 0.00 [0.00, 1.00] | 0.992 |
| Total weeks hospitalized (median [IQR]) | 0.00 [0.00, 0.50] | 0.00 [0.00, 2.00] | 0.483 |
| Total weeks hospitalized per year ill (median [IQR]) | 0.00 [0.00, 0.00] | 0.00 [0.00, 0.00] | 0.250 |
| Episodes of mania (median [IQR]) | 0.00 [0.00, 1.25] | 0.00 [0.00, 3.00] | 0.831 |
| Episodes of depression (median [IQR]) | 3.00 [1.00, 5.00] | 2.00 [1.00, 3.50] | 0.316 |
| Total number of episodes (median [IQR]) | 3.00 [1.00, 6.00] | 3.00 [1.00, 6.00] | 0.789 |

**Supplementary Table S16** : Biochemical parameters of study participants, by sex.

| **Variable** | **Female** | **Male** | **p-value** |
| --- | --- | --- | --- |
| Hematocrit (median [IQR]) | 40.90 [38.85, 42.63] | 44.80 [44.00, 47.30] | **<0.001** |
| Hemoglobin (median [IQR]) | 13.30 [12.65, 13.95] | 15.10 [14.61, 15.70] | **<0.001** |
| Erythrocytes (mean (SD)) | 4.57 (0.35) | 5.18 (0.47) | **<0.001** |
| MCV (median [IQR]) | 88.52 [85.00, 91.00] | 86.93 [84.28, 89.81] | 0.215 |
| MCH (median [IQR]) | 29.00 [27.81, 30.00] | 29.00 [28.54, 30.00] | 0.631 |
| MCHC (median [IQR]) | 32.89 [32.33, 33.50] | 33.48 [32.89, 33.98] | **0.007** |
| Leukocytes (median [IQR]) | 6.97 [5.60, 8.30] | 6.75 [5.40, 7.55] | 0.471 |
| Segmented neutrophils Percentage (median [IQR]) | 56.00 [52.00, 63.50] | 58.00 [50.00, 63.00] | 0.896 |
| Segmented neutrophils absolute count (median [IQR]) | 3.91 [2.93, 5.20] | 3.72 [2.97, 4.53] | 0.532 |
| Lymphocytes Percentage (mean (SD)) | 35.67 (8.77) | 35.08 (9.62) | 0.753 |
| Lymphocytes absolute count (median [IQR]) | 2.30 [1.94, 2.77] | 2.34 [1.87, 2.70] | 0.421 |
| Monocytes Percentage (median [IQR]) | 4.00 [2.00, 7.00] | 4.00 [3.00, 8.00] | 0.597 |
| Monocytes absolute count (median [IQR]) | 0.29 [0.18, 0.46] | 0.30 [0.19, 0.44] | 0.978 |
| Eosinophils Percentage (median [IQR]) | 2.00 [1.30, 2.10] | 2.00 [1.70, 3.00] | 0.671 |
| Eosinophils absolute count (median [IQR]) | 0.13 [0.10, 0.20] | 0.14 [0.10, 0.20] | 0.708 |
| Basophils Percentage (median [IQR]) | 0.00 [0.00, 0.00] | 0.00 [0.00, 0.00] | 0.928 |
| Basophils absolute count (median [IQR]) | 0.00 [0.00, 0.00] | 0.00 [0.00, 0.00] | 0.896 |
| ESR (median [IQR]) | 11.00 [10.00, 17.00] | 7.50 [5.00, 10.00] | **<0.001** |
| Urea (median [IQR]) | 27.00 [22.00, 34.00] | 33.00 [29.25, 40.75] | **<0.001** |
| Creatinine (median [IQR]) | 7.70 [0.94, 9.07] | 9.35 [1.12, 10.47] | **0.014** |
| GOT (median [IQR]) | 22.00 [16.50, 27.00] | 29.00 [22.00, 39.00] | **0.001** |
| GPT (median [IQR]) | 22.00 [14.00, 30.50] | 29.00 [22.00, 41.10] | **0.002** |
| Alkaline phosphatase (median [IQR]) | 154.00 [107.00, 190.00] | 145.00 [88.00, 184.00] | 0.626 |
| Total Bilirubin (median [IQR]) | 0.50 [0.38, 0.74] | 0.58 [0.49, 0.77] | **0.048** |
| Sodium (mean (SD)) | 137.97 (3.43) | 138.46 (3.42) | 0.485 |
| Potassium (median [IQR]) | 4.30 [4.00, 4.60] | 4.20 [4.00, 4.45] | 0.361 |
| Chloride (median [IQR]) | 96.00 [90.00, 100.00] | 96.00 [90.00, 100.00] | 0.904 |
| HS CRP (median [IQR]) | 1.36 [0.40, 4.12] | 1.10 [0.41, 3.34] | 0.625 |

**Supplementary Table S17** : Percentage of classical, intermediate, and non-classical monocytes in study participants, by sex.

| **Variable** | **Female** | **Male** | **p-value** |
| --- | --- | --- | --- |
| Classic monocytes (median [IQR]) | 79.30 [70.30, 85.20] | 81.95 [70.08, 85.85] | 0.791 |
| Intermediate monocytes (median [IQR]) | 8.66 [6.04, 13.70] | 9.43 [5.03, 15.07] | 0.754 |
| Non classic monocytes (median [IQR]) | 5.95 [3.48, 10.75] | 6.30 [3.97, 10.35] | 0.799 |

**Supplementary Table S18:** Plasma level of cytokines, chemokines and neurotrophic factors of study participants, by sex.

| **Variable** | **Female** | **Male** | **p-value** |
| --- | --- | --- | --- |
| CCL2 (median [IQR]) | 50.26 [38.26, 69.45] | 54.03 [34.99, 82.37] | 0.536 |
| sTREM2 (median [IQR]) | 889.79 [731.87, 1215.10] | 933.17 [667.91, 1204.14] | 0.776 |
| BDNF (median [IQR]) | 4673.30 [2792.94, 6793.28] | 4263.44 [2307.29, 6845.11] | 0.837 |
| IL-6 (median [IQR]) | 1.37 [0.90, 2.17] | 1.18 [0.86, 1.81] | 0.298 |
| β-NGF (median [IQR]) | 13.62 [5.27, 19.79] | 10.99 [4.50, 20.48] | 0.993 |
| IL-18 (median [IQR]) | 107.95 [33.21, 190.28] | 147.50 [68.36, 231.24] | 0.057 |
| TNFα (median [IQR]) | 45.90 [0.00, 103.47] | 24.34 [0.00, 101.74] | 0.726 |
| CX3CL1 (median [IQR]) | 685.88 [430.27, 1327.60] | 518.70 [423.17, 978.32] | 0.223 |
| IL-1β (median [IQR]) | 25.35 [13.17, 44.57] | 27.92 [7.94, 55.48] | 0.934 |
| IFNγ (median [IQR]) | 10.94 [5.95, 17.57] | 11.65 [7.30, 19.27] | 0.540 |
| CXCL8 (median [IQR]) | 12.65 [2.77, 32.40] | 11.79 [2.07, 60.73] | 0.708 |
| IL-10 (median [IQR]) | 6.12 [0.00, 18.08] | 5.99 [0.00, 11.10] | 0.767 |
| IL-12p70 (median [IQR]) | 6.73 [4.32, 11.53] | 6.68 [4.73, 10.31] | 0.878 |
| IL-17A (median [IQR]) | 1.49 [0.11, 3.40] | 2.02 [0.69, 2.85] | 0.873 |
| IL-23 (median [IQR]) | 9.53 [4.03, 17.34] | 9.43 [3.45, 16.36] | 0.780 |
| IL-33 (median [IQR]) | 54.52 [17.08, 97.12] | 59.07 [22.17, 111.60] | 0.729 |

**Supplementary Table S19** : T-cell values of study participants, by sex.

| **Variable** | **Female** | **Male** | **p-value** |
| --- | --- | --- | --- |
| Tcell CD3+CD4+ (median [IQR]) | 58.50 [52.45, 63.50] | 59.70 [49.40, 66.00] | 0.939 |
| Tcell CD3+CD8+ (median [IQR]) | 28.60 [25.50, 34.05] | 26.90 [23.70, 31.70] | 0.492 |
| Ratio CD4/CD8 (median [IQR]) | 1.98 [1.62, 2.43] | 2.09 [1.54, 2.62] | 0.710 |
| CD4+CD44+ (median [IQR]) | 44.50 [31.77, 63.42] | 52.30 [33.70, 61.80] | 0.570 |
| CD4+CD69+ (median [IQR]) | 2.39 [1.42, 4.56] | 3.00 [1.51, 6.23] | 0.467 |
| CD4+PD1+ (median [IQR]) | 5.03 [3.09, 8.99] | 7.22 [4.98, 12.10] | **0.048** |
| CD4+LAG3+ (median [IQR]) | 2.22 [1.09, 5.20] | 1.92 [0.98, 6.65] | 0.972 |
| Tregs CD4+CD25+ FOXP3+ (median [IQR]) | 3.58 [2.23, 7.26] | 5.46 [2.32, 8.11] | 0.421 |

**Supplementary Table S20**. Limit of Detection (LOD), Limit of Quantification (LOQ), and the Varianza intra-assay (CoV)**.**

| **Marker** | **LEGENDplex**  **Muli-Analyte**  **Flow Assay**  **Kit** | **Standard curve C1 to C7 (pg/mL)** | **LOD**  **(pg/mL)** | **LOQ**  **(pg/mL)** | **CoV**  **(intra**  **assay)** | **Range**  **(pg/mL)** | **% of**  **Non- detectable**  **(ND)** |
| --- | --- | --- | --- | --- | --- | --- | --- |
| **CCL2** | Cat. 740796 | 2.44 to 10000 | 1.18 | 3.89 | 2-3% | 16.25 – 243.7 | 0 % |
| **sTREM2** | Cat. 740796 | 6.10 to 25000 | 1.52 | 5.016 | 3-4% | 248.4 – 4262.0 | 0 % |
| **BDNF** | Cat. 740796 | 2.44 to 10000 | 0.95 | 3.13 | 2-6% | 756.4 – 17221.0 | 0 % |
| **β-NGF** | Cat. 740796 | 0.61 to 2500 | 0.27 | 0.89 | 4-6% | ND – 99.62 | 12.9 % |
| **IL-18** | Cat. 740796 | 2.44 to 10000 | 0.69 | 2.27 | 4-8% | ND – 888.3 | 9.5 % |
| **TNFα** | Cat. 740796 | 2.44 to 10000 | 0.82 | 2.70 | 6-7% | ND – 356.8 | 36.2 % |
| **CX3CL1** | Cat. 740796 | 97.65 to 400000 | 41.26 | 136.15 | 3-4% | 179.3 – 4738 | 0 % |
|  |  |  |  |  |  |  |  |
| **IL-1β** | Cat. 740809 | 2.44 to 10000 | 1.5 | 4.95 | 3.5-3.7% | ND – 136.1 | 5.2 % |
| **IFNγ** | Cat. 740809 | 2.44 to 10000 | 1.3 | 4.29 | 3.0-3.3% | ND – 53.37 | 12.1% |
| **CXCL8** | Cat. 740809 | 2.44 to 10000 | 2.0 | 6.6 | 2.9-3.0% | ND – 201.7 | 18.2 % |
| **IL-10** | Cat. 740809 | 2.44 to 10000 | 2.0 | 6.6 | 2.6-2.7% | ND – 81.47 | 33 % |
| **IL-12p70** | Cat. 740809 | 2.44 to 10000 | 2.0 | 6.6 | 3.2% | ND – 47.83 | 1.7 % |
| **IL-17A** | Cat. 740809 | 0.61 to 2500 | 0.5 | 1.65 | 2.1-2.4% | ND – 20.92 | 19.1 % |
| **IL-23** | Cat. 740809 | 2.44 to 10000 | 1.8 | 5.94 | 2.6-3.3% | ND – 58.84 | 3.4 % |
| **IL-33** | Cat. 740809 | 6.10 to 25000 | 4.4 | 14.52 | 3.6-4.3% | ND – 415.0 | 12.2 % |
|  |  |  |  |  |  |  |  |
| **Marker** | **ENZO**  **High sensitivity ELISA kit** | **Standard curve C1 to C7 (pg/mL)** | **Sensitivity (pg/mL)** |  |  | **Range**  **pg/mL** | **% of**  **Non- detectable**  **(ND)** |
| **IL-6** | Cat. 1780001 | 0.78 to 50 | 0.057 |  |  | 0.37–128.9 | 0 % |
|  |  |  |  |  |  |  |  |
| **Marker** | **Turbitest High sensitivity PCR Wiener lab.** | **Standard curve C1 to C7**  **(mg/L)** | **LOD**  **(mg/L)** | **LOQ**  **(mg/L)** | **CoV**  **(intra**  **assay)** | **Range**  **mg/L** | **% of**  **Non- detectable**  **(ND)** |
| **PCR** | Cat. 1008125 | 3.4 a 150 | 0.5 | 1.65 | 1.2-2.4 % | ND –70.06 | 8.1% |

Limit of Detection (LOD), Limit of Quantification (LOQ), and the Varianza intra-assay (CoV) were obtained from the manufacturer of each kit. Standard curves were run following the manufacturer´s instructions. The range with the minimal and maximum and the percentage of non-detectable (ND) values were obtained from the data measured in our assays. ND was imputed as 0.

**Supplementary Figure S1. Monocytosis in patients with MDE and LegendPlex system to measure simultaneously a panel of cytokines, chemokines, and neurotrophic factors by flow cytometry.**

**A)** Monocytosis was determined by comparing the absolute number and the percentage of monocytes in patients with active or remitted MDE vs HC. The percentage of Lymphocytes is also graphed and compared. Statistical differences among groups were calculated by Kruskal-Wallis non-parametric test, and pairwise comparisons were performed using the Holm-Bonferroni adjustment.

**B)** Representative dot plots showing beads A and B gating strategies based on SSC and FSC. The Internal fluorescence intensities in APC also discriminate additional beads. Each bead serves as the capture bead for a particular analyte in a biological sample. The customized Inflammatory panel B), including the determination of 8 analytes, and the neuroinflammation panel C), including the determination of 9 analytes. The fluorescent signal intensities in PE are proportional to the amount of bound analytes. The stándard curve is built based on the pre-determined concentration (C1, C3, C5, and C7) of standard analyte and the dynamic range on MFI values for PE to finally extrapolate the sample concentrations (pg/mL). The determination of free-TGFb and IL-6 was discarded since most value samples have a value of 0.

**Supplementary Figure S2**. Correlation analysis including clinical, biochemical, and immunological parameters using the Spearman test. The correlation between variables was evaluated using the Spearman correlation coefficient*
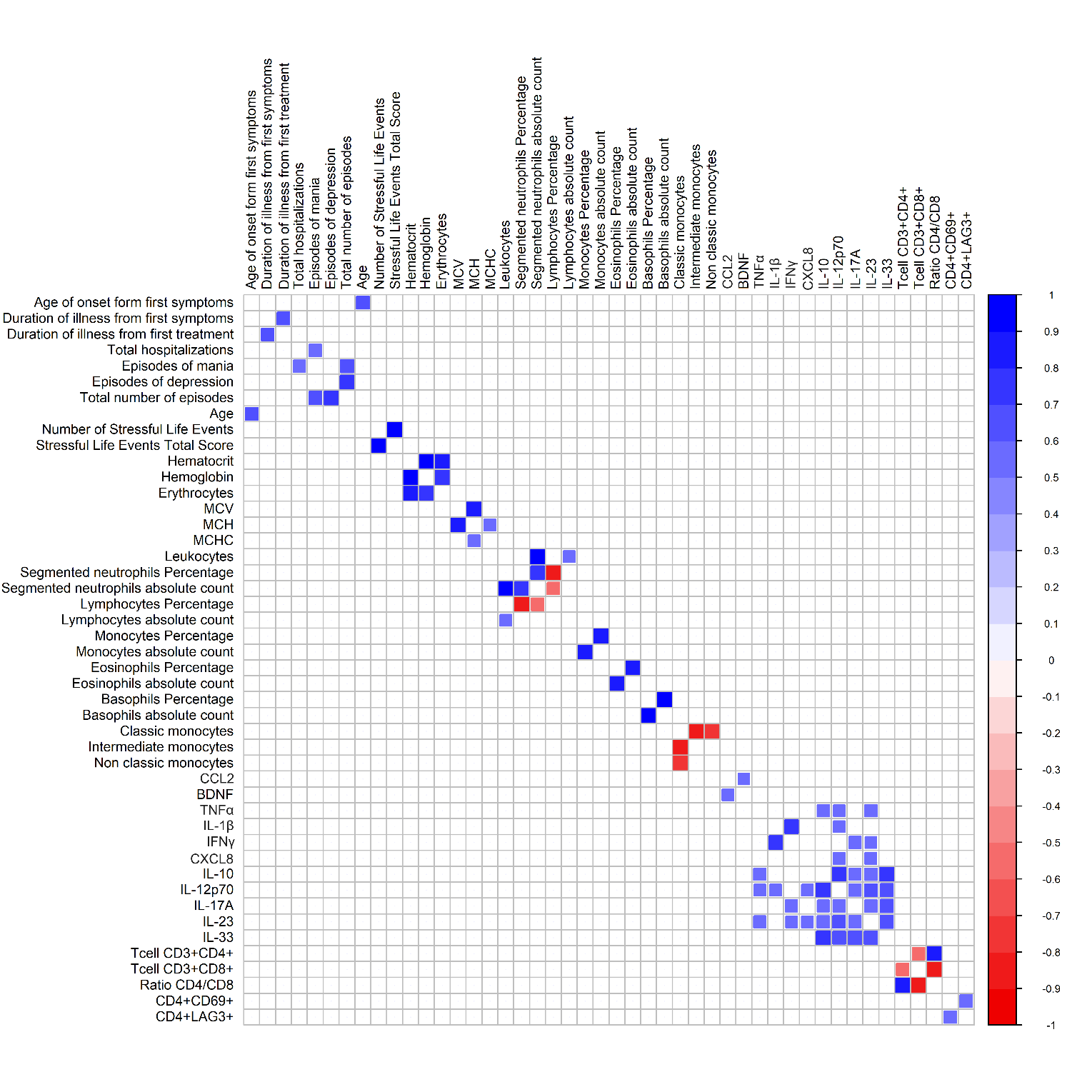
*
